# Neuronal Activity Promotes Node‐Like Cluster Assembly Prior to Myelination and Remyelination in the Central Nervous System

**DOI:** 10.1002/glia.70138

**Published:** 2026-02-23

**Authors:** Rémi Ronzano, Clément Perrot, Elisa Mazuir, Melina Thetiot, Marie‐Stéphane Aigrot, Paul Stheneur, François‐Xavier Lejeune, Bruno Stankoff, Catherine Lubetzki, Nathalie Sol‐Foulon, Anne Desmazières

**Affiliations:** ^1^ Sorbonne Université, Paris Brain Institute (ICM), INSERM U1127, CNRS UMR 7225, Hôpital de la Pitié‐Salpêtrière Paris France; ^2^ Data Analysis Core, Paris Brain Institute (ICM), INSERM U1127, CNRS UMR 7225, Sorbonne Université, Hôpital de la Pitié‐Salpétrière Paris France; ^3^ Département de Neurologie Assistance publique des Hôpitaux de Paris (APHP), Hôpital de la Pitié‐Salpétrière Paris France

## Abstract

In recent years, myelination has been recognized as a key process in neural plasticity and network refinement, in addition to its role in axonal conduction and neuronal metabolic support. The fast saltatory conduction along myelinated fibers relies on the nodes of Ranvier. Node‐like clusters can form prior to myelin deposition in development and repair and can regulate axonal conduction, myelin deposition initiation, as well as contribute to the formation of mature nodes of Ranvier. In this study, we show that node‐like cluster assembly is modulated by neuronal activity and could thus be an intermediate step in activity‐induced (re)myelination. We further identify the regulation of Nav1.1 expression, which we show to be required for node‐like cluster formation, as part of the molecular mechanism implicated in the activity‐dependent regulation of node‐like cluster assembly. This process may serve in shaping neuronal networks and myelin patterns during development, plasticity and repair.

## Graphical abstract

Ronzano et al. show that neuronal activity promotes node‐like cluster (NLC) formation before (re)myelination by regulating β1Nav and Nav1.1, which are essential for NLC assembly. This may represent an intermediate step in activity‐driven myelination and contribute to the regulation of axonal conduction.

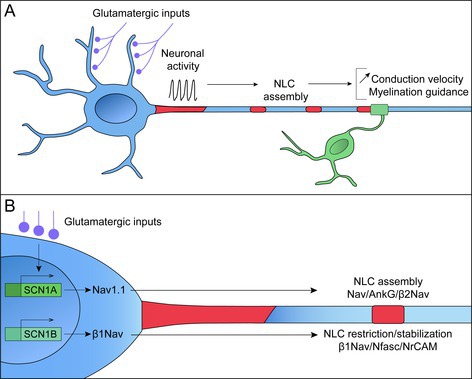

## 1 Introduction

Myelination enables rapid saltatory conduction along axons by alternating myelin‐insulated segments with the nodes of Ranvier. The nodes of Ranvier correspond to small amyelinic excitable domains highly enriched in voltage‐gated sodium channels (Nav) and potassium channels allowing the propagation of action potentials. In diseases such as multiple sclerosis, myelin loss, associated with nodal‐protein diffusion along axons, can lead to functional deficits and neuronal damage. The reorganization of myelinated fibers through remyelination can restore altered functions, as well as ensure neuroprotection (Lubetzki, Sol‐Foulon, and Desmazières 2020; Lubetzki, Zalc, et al. 2020). Nodal structures were classically described to assemble concomitantly with myelination (Amor et al. 2017; Pedraza et al. 2001; Rasband et al. 1999; Susuki et al. 2013). An alternative mechanism of assembly of node‐like clusters (or prenodes) has been characterized prior to myelin deposition in the central nervous system by our team and others and shown to depend on both oligodendroglial secreted cues and neuronal intrinsic factors (Bonetto et al. 2019; Dubessy et al. 2019; Elbaz et al. 2024; Freeman et al. 2015; Hildebrand and Waxman 1984; Kaplan et al. 1997, 2001; Thetiot et al. 2020; Vagionitis et al. 2022; Waxman et al. 1982). Node‐like clusters, which correspond to isolated clusters of nodal markers not flanked by paranodes or myelin, have been described along the axon of various neuronal subtypes, including parvalbumin and somatostatin GABAergic neurons of the hippocampus, cerebellar Purkinje cells and retinal ganglion cells (Bonetto et al. 2019; Dubessy et al. 2019; Freeman et al. 2015; Kaplan et al. 1997, 2001; Lubetzki, Sol‐Foulon, and Desmazières 2020; Thetiot et al. 2020; Vagionitis et al. 2022). These structures are observed in all the vertebrate species examined, namely zebrafish, mouse, rat, and human (Coman et al. 2006; Lubetzki, Sol‐Foulon, and Desmazières 2020; Vagionitis et al. 2022). Interestingly, they can form along both glutamatergic or GABAergic neurons, and appear to be more specifically associated with long‐projecting or highly complex axons (Lubetzki, Sol‐Foulon, and Desmazières 2020). In multiple sclerosis, similar structures have been reported in remyelinating lesions (Coman et al. 2006).

Functionally, node‐like clusters have proved to accelerate axonal conduction velocity, possibly through micro‐saltatory conduction (Freeman et al. 2016; Neishabouri and Faisal 2014). They further participate in heminodal and mature node assembly and guide myelination initiation at their direct vicinity (Freeman et al. 2015; Malavasi et al. 2021; Thetiot et al. 2020; Vagionitis et al. 2022). In the zebrafish, a subset of node‐like clusters is thought to predefine mature nodes of Ranvier localization (Vagionitis et al. 2022), suggesting that they could be landmarks for axonal domain and myelination pattern organization. It is thus of importance to better understand the mechanisms regulating node‐like cluster formation in health and repair.

Neuronal activity is known to play a key role in the induction of developmental myelination (Almeida et al. 2021; Barres and Raff 1993; Braaker et al. 2025; Demerens et al. 1996; Geraghty et al. 2019; Gibson et al. 2014; Hines et al. 2015; McKenzie et al. 2014; Mensch et al. 2015; Mitew et al. 2018; Wake et al. 2011), as well as modulate remyelination under pathological conditions (Bacmeister et al. 2020; Gautier et al. 2015; Ortiz et al. 2019). It further participates in adaptive myelination, which allows the fine tuning of neuronal networks in different areas of the central nervous system (CNS) to promote coordinated activity and memory consolidation (Etxeberria et al. 2016; Pan et al. 2020; Steadman et al. 2020; Teissier et al. 2020). Of note, these previous studies explored how neuronal activity influences myelination and remyelination per se, but did not unravel its effects on axonal microdomain organization.

Here, we have investigated in various neuronal subpopulations whether neuronal activity regulates the assembly of node‐like clusters prior to myelin deposition. First, we have modulated neuronal activity and glutamatergic excitatory neurotransmission in mixed hippocampal cultures, where node‐like clusters form along the axons of GABAergic neurons. In this context, neuronal activity promotes node‐like cluster assembly, whereas the inhibition of glutamatergic receptors leads to its reduction. The inhibition of glutamatergic signaling is further associated with a decreased expression of some nodal proteins, including the voltage‐gated sodium channel isoform Nav1.1, which we show to be critical for node‐like clustering. The role of neuronal activity in node‐like cluster (re)assembly has further been confirmed using chemogenetic and optogenetic stimulations along Purkinje axons ex vivo. Lastly, neuronal activity also promotes node‐like cluster reassembly at the onset of remyelination in vivo in mouse spinal cord, further strengthening the physiological relevance of this process.

## 2 Results

### 2.1 Neuronal Activity Modulates Node‐Like Cluster Formation Along the Axon of GABAergic Neurons In Vitro

In mixed hippocampal cell cultures, node‐like clusters (i.e., isolated clusters not flanked by paranodal structure(s) or myelin) are first observed around 14 days in vitro (DIV) on a subset of GABAergic neurons, with the percentage of GABAergic neurons with node‐like clusters increasing over time (Freeman et al. 2015). In these cultures, neurons are electrically active, with GABAergic neurons firing action potentials (APs) of short duration and receiving high frequencies of glutamatergic excitatory synaptic events (Mazuir et al. 2021).

**FIGURE 1:**
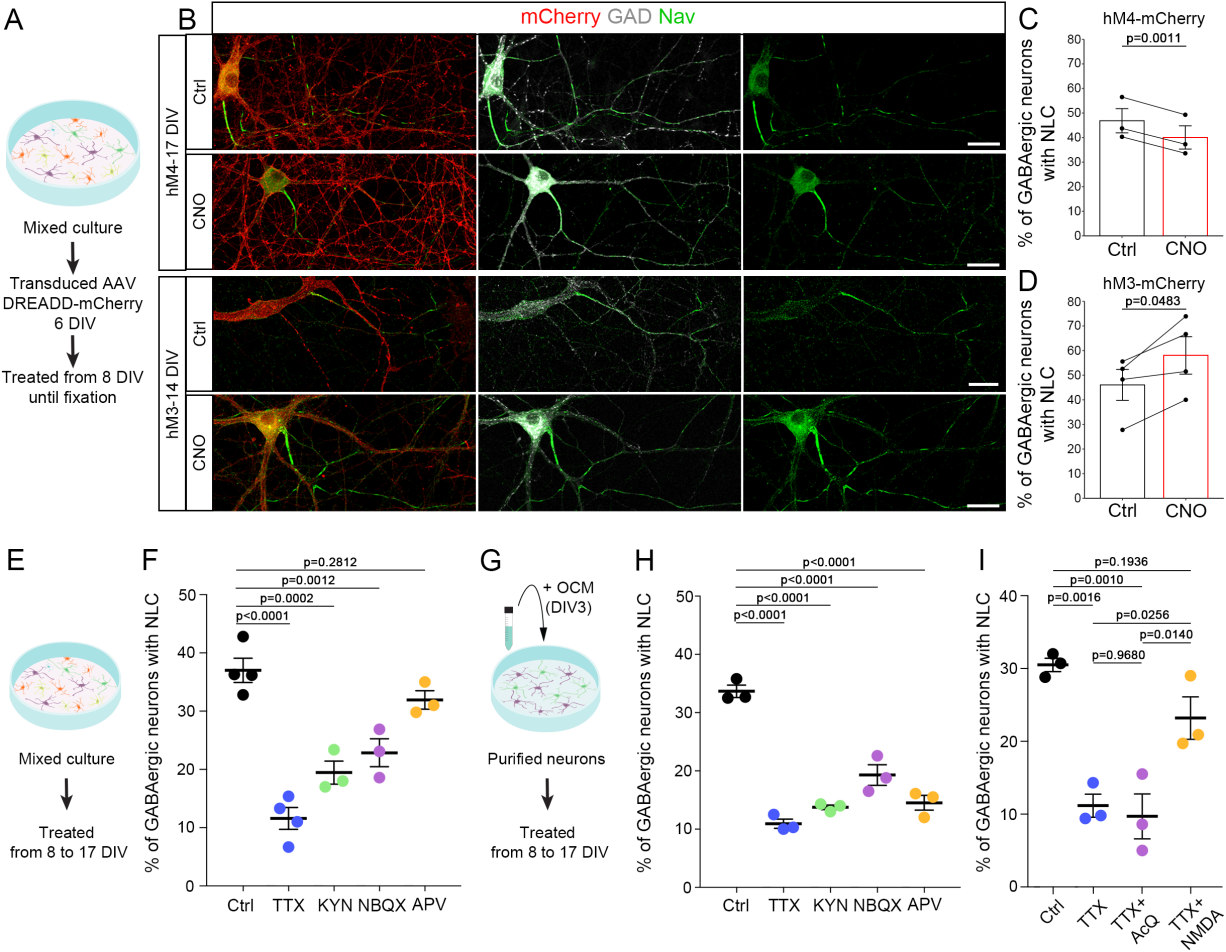
Node‐like cluster assembly along the axon of GABAergic neurons is modulated by neuronal activity and glutamatergic inputs. (A) Neuronal activity was modulated by expression of the DREADDs receptor hM4D(Gi)‐mCherry or hM3D(Gq)‐mCherry in neurons in mixed hippocampal cultures, followed by the addition of the DREADDs ligand N‐Clozapine (CNO, 0.5 μM) to the cultures, while control cultures were treated with DMSO only. (B) Illustration of GABAergic neurons (GAD67+, white) expressing DREADDs receptor hM4D(Gi)‐mCherry or hM3D(Gq)‐mCherry (mCherry+, red) with node‐like clusters along their axons (Nav, green) in control condition or following CNO treatment. (C, D) Percentage of transduced hM4D(Gi) (C) and hM3D(Gq). (D) GAD+ neurons with node‐like clusters in control condition or following CNO treatment. The values plotted individually correspond to the percentage obtained for the different experiments (hM4D(Gi), *n* = 3 experiments; hM3D(Gq), *n* = 4 experiments). (E–H) Mixed hippocampal cultures (E) or purified neuron cultures supplemented with oligodendrocyte conditioned medium (OCM) (G) were treated with TTX (0.1 μM) or glutamatergic antagonists (kynurenic acid, KYN, 1 mM; NBQX, 10 μM or APV, 100 μM). (F, H) Percentage of GAD+ neurons with node‐like clusters following treatment by TTX, KYN, NBQX or APV in mixed primary culture (F) and purified neuron culture treated with OCM (H). (I) Percentage of GAD+ neurons with node‐like clusters following treatment with TTX (0.1 μM) or TTX and glutamatergic agonists (NMDA (20 μM) or quisqualic acid (AMPA/Kainate and group I metabotropic glutamate receptors, 1 μM)) in purified neuron cultures supplemented with OCM (*n* = 3 experiments). (C, D) Two‐sided paired t‐tests. (F, H, I) One‐way ANOVA followed by Dunnett's multiple comparison test. Scale bars: 30 μm.

To assess whether node‐like cluster formation could be regulated by neuronal activity, we first used AAV transduction of mixed hippocampal cell cultures to express the DREADDs receptor hM3D(Gq)‐mCherry (activation) or hM4D(Gi)‐mCherry (inhibition) under the control of the pan‐neuronal human synapsin promoter. We then assessed the percentage of GAD+/mCherry+ neurons with node‐like clusters, following treatment with the DREADDs activator N‐Clozapine (CNO, 0.5 μM) from 8 DIV until fixation of the cultures, compared to control condition (Ctrl, Figure 1A).

The inhibition of neuronal activity mediated by hM4D(Gi)‐mCherry led to a small but significant decrease in GAD+ mCherry+ neurons with node‐like clusters (Figure 1B,C), whereas the activation of neuronal activity mediated by hM3D(Gq)‐mCherry led to a 25% increase in the population of GAD+ mCherry+ neurons with node‐like clusters (Figure 1B,D). These results show that neuronal activity promotes node‐like cluster assembly in GABAergic neurons in vitro. Furthermore, the distance between node‐like clusters was significantly reduced by 25% following neuronal activity inhibition, with a tendency toward an increased distance following neuronal activity reinforcement, but no significant variation in node‐like cluster length (see statistics table).

### 2.2 The Pharmacological Inhibition of Neuronal Activity and Glutamatergic Signaling Leads to Reduced Node‐Like Cluster Assembly In Vitro

The DREADDs strategy is based on GPCR (G‐Protein coupled receptors), which upon activation can also lead to the activation of Gi and Gq canonical pathways, in addition to neuronal activity modulation (Roth 2016; Sternson and Roth 2014). To confirm the direct role of neuronal activity on the regulation of node‐like clusters, we used tetrodotoxin (TTX), a toxin blocking Na+ ion flux through the Nav channel, thereby preventing AP generation and propagation. The addition of TTX in mixed hippocampal cultures from 8 DIV to 17 DIV (Figure 1E) led to a 69% reduction in the percentage of GABAergic neurons with clusters compared to control condition (Figure 1F). To exclude that the modulation of node‐like clusters could be due to non‐autonomous cellular mechanisms mediated through the glial cells, and in particular to the oligodendrocytes present in the culture, we repeated these experiments on purified hippocampal neurons treated with oligodendrocyte conditioned medium (OCM; Freeman et al. 2015). A similar effect of TTX was observed under this condition compared to mixed hippocampal cultures, with a strong decrease in node‐like cluster assembly (Figure 1G,H). Thus, neuronal activity can modulate node‐like cluster formation by directly regulating neuronal properties.

We next determined the effect of excitatory glutamatergic signals on node‐like cluster formation. We used different antagonists of glutamatergic receptors that is kynurenic acid (KYN), which inhibits alpha‐amino‐3‐hydroxy‐5‐methyl‐4‐isoxazole propionic acid (AMPA), kainate, and *N*‐methyl‐d‐aspartate (NMDA) receptors, as well as NBQX and APV, which are selective inhibitors of AMPA/kainate and NMDA receptors respectively. Adding KYN induced a 2‐fold reduction in clustering activity in mixed cultures and OCM treated cultures (Figure 1F,H). These data show that neuronal glutamatergic receptors participate in node‐like clustering modulation.

Similar results were observed using NBQX, but interestingly, APV induced a significant reduction of clustering activity on purified hippocampal neurons treated with OCM but not on mixed cultures.

To further explore the neuronal autonomous regulation of this process, we asked whether NMDA or Quisqualic acid (AcQ, an agonist of the AMPA/Kainate and group I metabotropic glutamate receptors) could rescue node‐like cluster formation following neuronal activity inhibition by TTX in purified neuron cultures treated with OCM. We confirmed the strong decrease in cluster assembly in TTX compared to control condition (Figure 1I). We further observed that NMDA addition could partially rescue node‐like clustering in a significant manner, while AcQ could not (Figure 1I).

These data suggest a prominent role of NMDA receptors in the neuronal autonomous regulation of node‐like cluster assembly.

### 2.3 Inhibition of Glutamatergic Neurotransmission Alters the Expression of Specific Nodal Proteins in Hippocampal GABAergic Neurons

Node‐like clusters contain the main markers of nodes of Ranvier, such as Nav channels (Nav1.1, Nav1.2, with Nav1.6 expression being more sparse or absent), associated with β1Nav and β2Nav subunits, Nfasc186, NrCAM, and Ankyrin G (AnkG) (Bonetto et al. 2019; Dubessy et al. 2019; Freeman et al. 2015; Kaplan et al. 1997; Thetiot et al. 2020). A strong diffuse expression of Caspr and Kv1.2 has been observed along axons with node‐like clusters (Bonetto et al. 2019), but whether—and which—nodal potassium channels could be clustered at the node‐like structures has not yet been investigated. It was, however, shown by patched single cell‐RNA sequencing that *KCNQ2/3* (Kv7.2/3) and *KCNC1* (Kv3.1b) are expressed in node‐like forming hippocampal GABAergic neurons in culture, and it is described that all three can be found at mature nodes (Lubetzki, Sol‐Foulon, and Desmazières 2020; Mazuir et al. 2021).

As glutamatergic pathways, and in particular the ones relying on NMDA receptors, are known to modulate gene expression, we first asked whether glutamatergic signals could impact node‐like cluster assembly through the modulation of nodal protein expression. For this purpose, we quantified the percentage of GABAergic neurons expressing various proteins known to be present at the axon initial segment (AIS) and at node‐like clusters in GABAergic neurons, comparing mixed hippocampal cultures treated with kynurenic acid (KYN) with the control condition.

**FIGURE 2:**
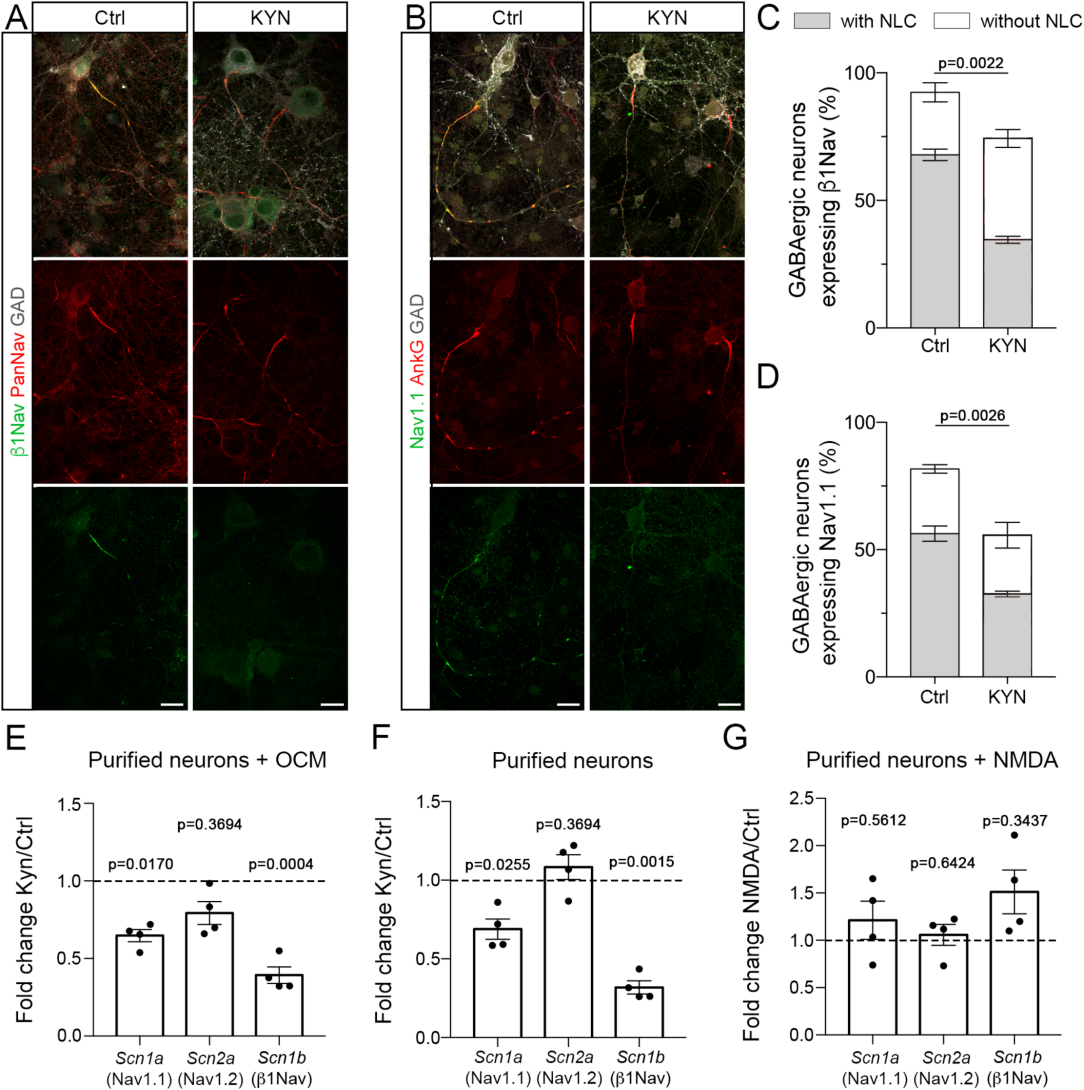
Glutamatergic inputs modulate specific nodal marker expression in GABAergic neurons. The inhibition of glutamatergic inputs in mixed hippocampal cultures by the addition of a glutamatergic antagonist (kynurenic acid, KYN, 1 mM) affects the expression in GABAergic neurons (GAD+, white) of β1Nav (A, C), Nav1.1 (B, D). (C, D) Percentages of GAD+ neurons expressing the nodal marker of interest (percentage of cells with node‐like clusters in dark and of cells without clusters in white) in control condition or following treatment by KYN. Unpaired *t* test. Scale bars: 20 μm. (E–G) Expression of Scn1a, Scn2a, and Scn1b transcripts in purified hippocampal cultures with OCM (E) or without OCM (F, G), in control condition or following treatment by KYN (E–F) or NMDA (G). Gene expression was determined by RT‐qPCR and relative expression compared to control were plotted (*n* = 4 experiments). (E–G) One‐sample *t* test followed by correction using FDR method.

We first observed that AnkG and PanNav stainings are always present in GABAergic neurons, at least at the AIS, independently of the condition considered (Figures 2A,B and S1A–D). GABAergic cells can express Nav1.1, Nav1.2, and Nav1.6 isoforms at node‐like clusters and axon initial segments, while Nav1.1 is absent in glutamatergic hippocampal neurons (Freeman et al. 2015). We assessed the expression of Nav1.1, as well as of Neurofascin (Nfasc), Nav subunits β1Nav and β2Nav, and the potassium channels Kv7.2 and Kv3.1b in GABAergic neurons in control condition and following KYN treatment.

While β2Nav, Nfasc, and Kv7.2 were expressed in the vast majority of GABAergic neurons in control and KYN condition (Figure S1B–D,F–H), the percentages of GABAergic neurons expressing Nav1.1, β1Nav, and Kv3.1b were significantly decreased following KYN treatment (Figures 2A–D and S1A,E).

The decreased expression of Nav1.1, β1Nav, and Kv3.1b following kynurenic acid treatment was linked to a loss of expression of these proteins in a subpopulation of GABAergic neurons deprived of node‐like clusters, while their expression was preserved in most GABAergic neurons with clusters (Figure S1I–K).

To further confirm this mechanism and explore its neuronal cell‐autonomous aspect, we performed RT‐qPCR following KYN treatment compared to control condition, on purified neuron cultures with or without OCM (Figure 2E,F respectively). We observed a significant reduction of both *Scn1a* (encoding Nav1.1) and *Scn1b* (encoding β1Nav) expression, but not *Scn2a* (encoding Nav1.2) for both conditions (Figure 2E,F). While *Scn2a* expression was unchanged, we further observed a tendency toward an increased expression of *Scn1a* and *Scn1b* following NMDA treatment of purified neurons (Figure 2G), suggesting a role of NMDA receptors in the modulation of *Scn1a* and *Scn1b* expression by glutamatergic inputs.

We further assessed whether node‐like clustering could also be reduced following glutamatergic signal alteration through the alteration of nodal marker axonal transport. We expressed the nodal markers β1Nav or β2Nav coupled to mCherry by transfection of mixed hippocampal cultures and imaged the axonal transport of the mCherry+ puncta by videomicroscopy at 16–17 DIV as previously described (Thetiot et al. 2020). We then generated kymographs representing puncta axonal trajectories along time (Figure S1L). For both markers, the mean number of puncta per 100 μm and the distribution of puncta amongst various categories (anterograde, retrograde or bidirectional movement, as well as non‐moving puncta) showed no significant differences between KYN and control conditions (Figure S1M–P).

These results suggest that glutamatergic inputs can modulate specifically some nodal protein expression in hippocampal GABAergic neurons, which in turn could lead to a variation of node‐like cluster assembly in these cells.

### 2.4 Nav1.1 is required for efficient node‐like cluster assembly in GABAergic neurons in vitro

Amongst the nodal proteins observed to be modulated by glutamatergic inputs, Nav1.1 was the only one consistently expressed in all node‐like clusters observed along GABAergic axons (Figure 2B; Freeman et al. 2015; Thetiot et al. 2020).

**FIGURE 3:**
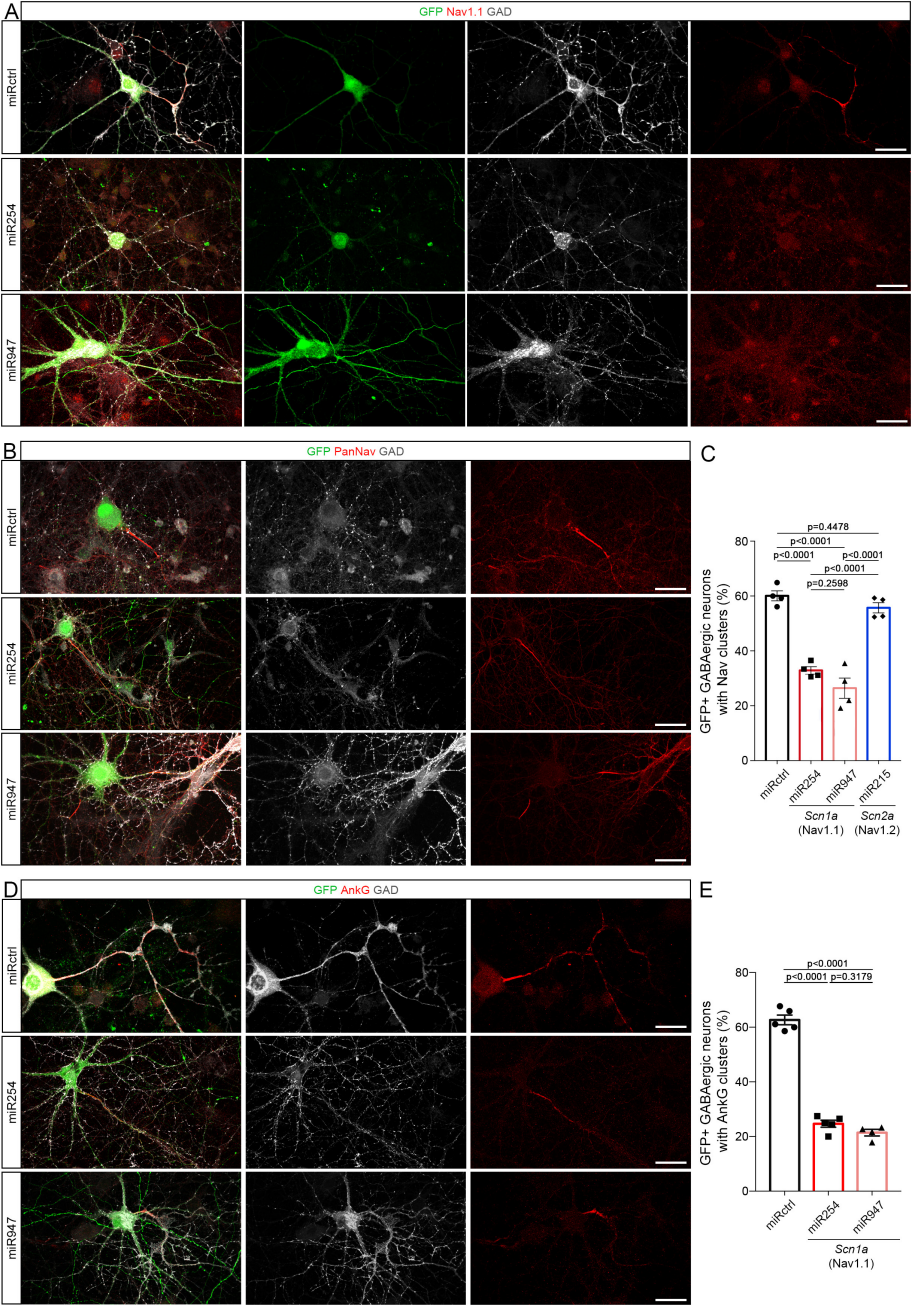
Nav1.1 downregulation leads to decreased node‐like cluster formation. (A) Both *Scn1a*‐targeting miRNA (miR254 and miR947) lead to an efficient downregulation of Nav1.1 expression (in red), compared to control miRNA (miRCtrl), in transfected GABAergic neurons (GAD+ neurons, in white, expressing emGFP, in green). (B, D) Node‐like cluster assembly is reduced in GABAergic neurons expressing *Scn1a* miRNA compared to control miRNA, as seen with Nav (B, in red) and AnkG stainings (D, in red). (C, E) Percentage of GAD+ transfected neurons with node‐like clusters, assessed using PanNav (C) or AnkG (E). The percentage of neurons with node‐like clusters is significantly reduced following *Scn1a* downregulation (miR254 and 947) compared to control and *Scn2a* downregulation (miR215). (C, E) One‐Way ANOVA, followed by Dunnett's multiple comparison test. Scale bars: 30 μm.

This led us to assess whether Nav1.1 expression was required to efficiently assemble node‐like clusters in vitro. We thus designed two miRNA (miR254 and miR947) specifically targeting *Scn1a* (which encodes Nav1.1) to be compared with a control miRNA and a *Scn2a*‐targeting miRNA (miR215). All miRNA were coupled to emGFP expression in order to ensure the detection of the transfected neurons. We first validated the efficiency and specificity of miR254, 947, and 215 using an RNAscope approach to detect *Scn1a* and *Scn2a* mRNA (Figure S2A,B, respectively). Interestingly, the downregulation of *Scn1a* expression by miR254 and 947 was associated with an increase in *Scn2A* expression (Figure S2B). We next confirmed that Nav1.1 channel expression was lost following the expression of the *Scn1a*‐targeting miRNA in GABAergic neurons, while it was still expressed in GABAergic neurons expressing the control construct (Figure 3A). We then quantified the percentage of emGFP+ GABAergic neurons with node‐like clusters at 17 DIV, comparing the two *Scn1a*‐targeting miRNAs with the control construct or *Scn2a* targeting miRNA (Figures 3B–E and S3), using PanNav staining to detect the clusters. We observed a strong and significant decrease in the percentage of GABAergic cells with node‐like clusters when miRNA miR254 or 947 were expressed compared to control condition (Figure 3B,C, 45% and 61% decrease respectively), while node‐like clustering was not affected by miR215 expression (Figures 3C and S3). This was confirmed by further using AnkG to quantify node‐like clusters in the presence of miR254 or 947 (Figure 3D,E, 61% and 66% decrease respectively) compared to the control miRNA.

These results show that Nav1.1 is required for efficient node‐like cluster assembly in a specific manner. Altogether, our data support the idea that neuronal activity impacts node‐like cluster formation through the modulation of Nav1.1 expression by glutamatergic inputs and more specifically by NMDA receptor activation.

### 2.5 Node‐Like Cluster Assembly is Modulated by Neuronal Activity Prior to Myelination in the Cerebellum Ex Vivo

As mentioned above, node‐like clusters are not restricted to hippocampal GABAergic neurons but can be transiently present prior to myelin deposition in various subsets of neurons. We in particular observed the assembly of such structures along cerebellar Purkinje cells during developmental myelination in vivo, as well as in cultured cerebellar slices ex vivo (Figure S4A,B,D,E), with a similar density at the onset of myelination and remyelination (Figure S4F), but a significantly increased density in remyelination compared to isolated Nav clusters observed in demyelinated slices (Figure S4G–I).

To assess whether the modulation of node‐like cluster formation by neuronal activity is a general feature, we developed two approaches to modulate neuronal activity in Purkinje cells using organotypic cultures of cerebellar slices.

We first expressed hM3D(Gq)‐mCherry by AAV transduction of cerebellar slices and treated the slices with N‐Clozapine at the very onset of myelination. We first confirmed that this strategy allowed a significant increase in neuronal activity by patch clamp of mCherry^+^ Purkinje cells (Figure S5A). The recordings performed showed a 4‐fold increase in Purkinje cell discharge frequencies following CNO addition to the medium (Figure S5B,C).

**FIGURE 4:**
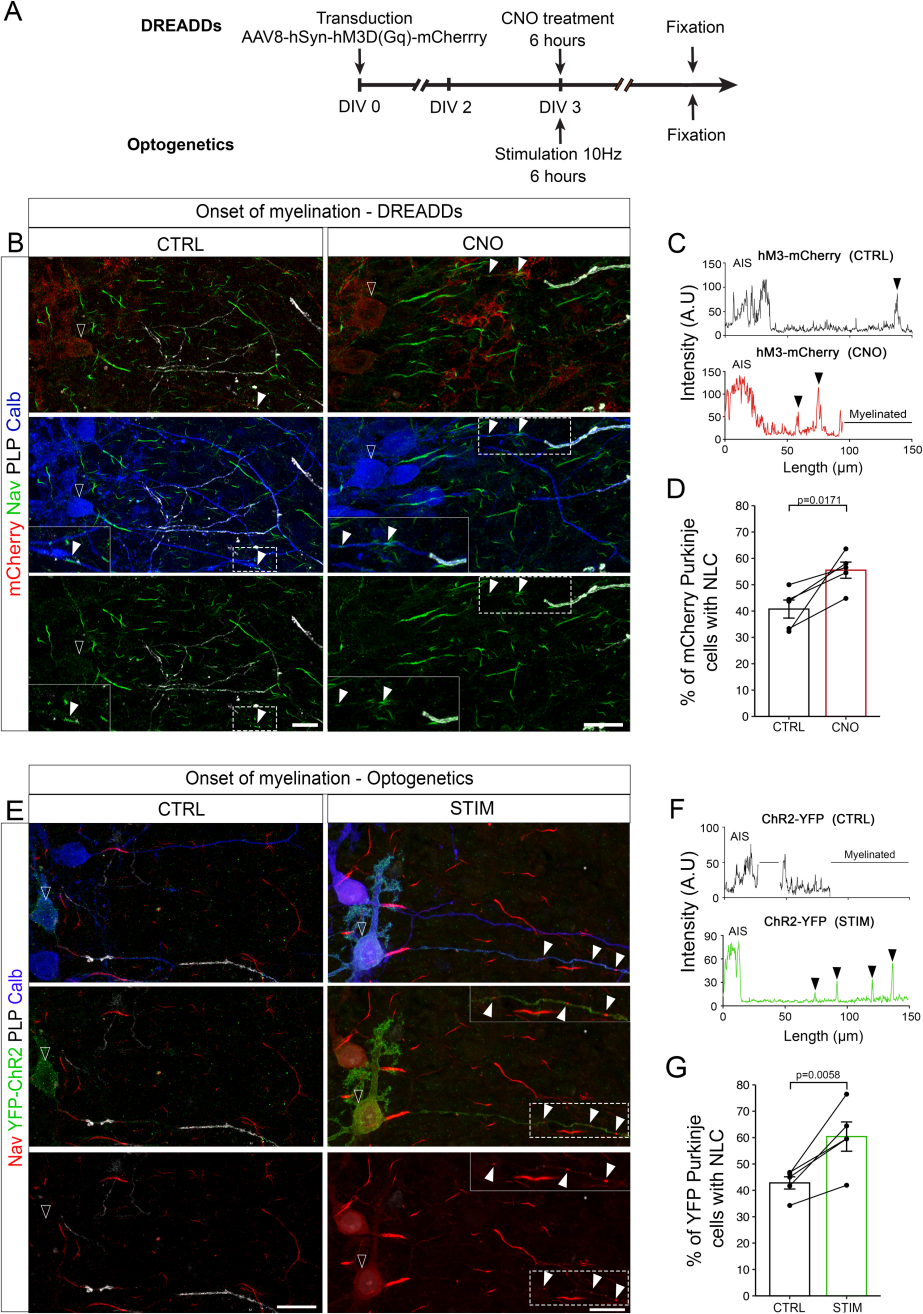
Neuronal activity enhances node‐like cluster assembly along Purkinje cells prior to myelination. For the DREADDs approach, cerebellar slices were transduced with AAV8‐hSyn‐hM3D(Gq)‐mCherry after being generated, treated with CNO or DMSO (Ctrl) at 3 DIV for 6 h and fixed. For the optogenetics approach, L7‐ChR2‐YFP mouse cerebellar slices were illuminated (470 nm, STIM) or not (Ctrl) for 6 h at 3 DIV, before being fixed. (B) Purkinje cells (Calbindin, blue) transduced with DREADDs‐expressing AAV (mCherry+, red, contour arrowhead) with node‐like clusters along their axon (Nav, green, filled arrowhead) in control or CNO‐treated slices. (C) Plot profile of Nav staining intensity along mCherry+ Purkinje cell axons (contour arrowhead) in control (Ctrl) or CNO condition shown in B. Arrowheads show node‐like clusters. (D) Percentage of mCherry+ Purkinje cells with node‐like clusters following CNO (0.5 μM) or DMSO treatment (Ctrl). (E) Purkinje cells (Calbindin, Blue) expressing ChR2 (YFP, Green, white contour arrowhead) with node‐like clusters along their axon (Nav, red, white filled arrowhead). Arrowheads indicate node‐like clusters. (F) Plot profile of Nav staining intensity along YFP+ Purkinje cell axons in control and stimulated condition shown in E. (G) Percentage of YFP+ Purkinje cells with node‐like clusters following 6 h of stimulation (470 nm, STIM) or in control condition (CTRL). (D) *n* = 5 animals, Paired t test. (G) *n* = 5 animals, Paired t‐test. (B, E) Scale Bar: 20 μm.

We next assessed whether DREADD‐mediated increase in neuronal activity could modulate node‐like cluster formation at the onset of myelination (3 DIV; Figure 4A,B). We indeed observed a 1.4‐fold increase in the percentage of mCherry^+^ Purkinje cells with node‐like clusters following the reinforcement of their firing activity (Figure 4C,D).

To confirm these results and reinforce the neuronal specificity of our approach, we further developed a custom‐designed optogenetics set‐up allowing the controlled illumination at a wavelength of 470 nm of cerebellar slices over time (See Section 4 for detailed description). We used this set‐up to stimulate organotypic cultures of cerebellar slices generated from L7‐ChR2‐YFP mice (Chaumont et al. 2013), which allows us to specifically modulate Purkinje cell activity though the expression of a channelrhodopsin (ChR2) under control of the L7/pcp2 promoter. We first assessed the percentage of Purkinje cells expressing ChR2‐YFP in the slices at the onset of myelination (Figure S5D–F, around 80% of YFP expressing Purkinje cells in the folia analyzed in our study). We next used an illumination pattern of 10 ms pulses at 10 Hz to induce a significant increase in Purkinje cells firing while staying within their physiological firing range (Figure S5G–I; Van Der Heijden et al. 2021).

We then assessed whether an increase in neuronal activity induced by optogenetics in Purkinje cells could modulate node‐like cluster assembly at the onset of myelination (6‐h stimulation at 3 DIV, Figure 4A,E). Optogenetics stimulation led to a significant increase in the percentage of YFP+ Purkinje cells with node‐like clusters in stimulated compared to control cells (Figure 4F,G).

To assess whether neuronal activity enhancement could later on affect myelination along Purkinje cell axons ex vivo, we further performed the same activation paradigms using DREADDs or optogenetics at 3 DIV (Figure S6B–E, respectively), but fixed the slices the following day, when Purkinje cell axons are actively being myelinated. We quantified the rate of myelination in activated versus control slices (Figure S6) and observed a significant increase of myelination along Purkinje cell axons for both approaches.

Finally, we assessed whether altering glutamatergic input signaling could reduce node‐like cluster formation along Purkinje cell axon at the onset of myelination, as previously observed for hippocampal GABAergic neurons. Organotypic cerebellar slices were exposed to kynurenic acid (KYN) and Nav1.1 expression, as well as node‐like clustering, were assessed in Purkinje cells (Figure S7). We observed a decrease in the percentage of Purkinje cells expressing Nav1.1 (Figure S7A,B), and a small but significant decrease in the percentage of Purkinje cell axons with node‐like clusters following KYN treatment (Figure S7E,F), thus supporting the observations made in vitro.

### 2.6 Node‐Like Cluster Assembly Depends on Neuronal Activity During Remyelination in the Cerebellum Ex Vivo

Organotypic cerebellar slice cultures myelinate spontaneously, with the vast majority of Purkinje cell axons being myelinated, which allows to induce demyelination in these cultures using lysophosphatidylcholine (LPC) and study spontaneous remyelination of the demyelinated fibers ex vivo (Birgbauer et al. 2004; Thetiot et al. 2019). Node‐like clustering was observed in the demyelinated slices just prior to remyelination, with a similar density to the one observed ex vivo during myelination (Figure S4C,F).

**FIGURE 5:**
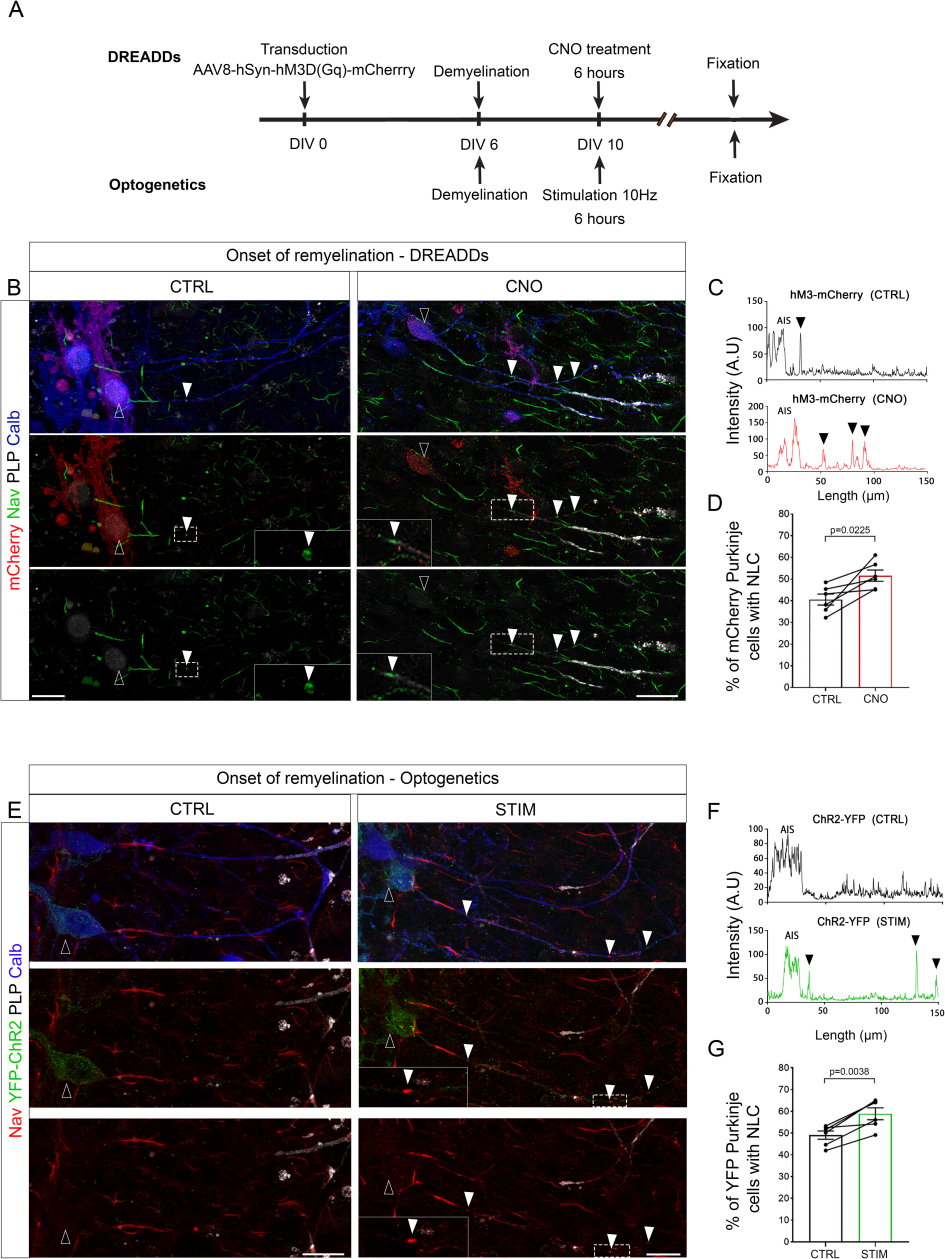
Neuronal activity enhances node‐like cluster assembly along Purkinje cells prior to remyelination. For the DREADDs approach, cerebellar slices were transduced with AAV8‐hSyn‐hM3D(Gq)‐mCherry after being generated, demyelinated at 6 DIV (LPC, 0.5 mg/mL, overnight treatment). They were next treated with CNO (0.5 μM) or DMSO (Ctrl) at DIV10 and fixed 6 h later. For the optogenetics approach, L7‐YFP‐ChR2 mouse cerebellar slices were demyelinated at 6 DIV (LPC, 0.5 mg/mL, overnight treatment) and illuminated (470 nm, STIM) or not (Ctrl) for 6 h at 10 DIV, before being fixed. (B) Purkinje cells (Calbindin, blue) expressing hM3D(Gq)‐mCherry (mCherry+, red, contour arrowhead) with node‐like clusters along their axon (Nav, green, filled arrowheads) in control or CNO‐treated slices. (C) Plot profile of Nav staining intensity along mCherry+ Purkinje cell axons in control (Ctrl) or CNO condition in B. Arrowheads indicate node‐like clusters. (D) Percentage of mCherry+ Purkinje cells with node‐like clusters following CNO (0.5 μM) or DMSO treatment (Ctrl). (E) Purkinje cells (Calbindin, blue) expressing ChR2 (YFP+, green, contour arrowhead) with node‐like clusters (Nav, red, filled arrowheads) along their axon. (F) Plot profile of Nav staining intensity along YFP+ Purkinje cell axons from E, in the control and activated condition. Arrowheads indicate node‐like clusters. (G) Percentage of YFP+ Purkinje cells with node‐like clusters following 6 h of stimulation (470 nm, STIM) or in control condition (CTRL). (D, G) Each point corresponds to one animal. (D) *n* = 6, Paired t test. (G) *n* = 6, Paired *t* test. (B, E) Scale Bar: 20 μm.

We took advantage of this approach to next assess whether neuronal activity could further modulate node‐like clustering during remyelination. Neuronal activity was increased using the DREADDs approach at the onset of remyelination (10 DIV, Figure 5A,B). We observed a 1.3‐fold increase of the percentage of mCherry^+^ Purkinje cells with node‐like clusters following the reinforcement of their activity (Figure 5C,D).

We next used the optogenetics stimulation, as described above, at the onset of remyelination (10 DIV, Figure 5A,E). This led to a significant increase in the percentage of YFP+ Purkinje cells with node‐like clusters in illuminated compared to control cells (Figure 5F,G). Altogether, these results show that neuronal activity can promote node‐like cluster assembly at the onset of remyelination, similarly to what is observed during developmental myelination.

### 2.7 Node‐Like Cluster Reassembly Is Modulated by Neuronal Activity During Remyelination In Vivo

**FIGURE 6:**
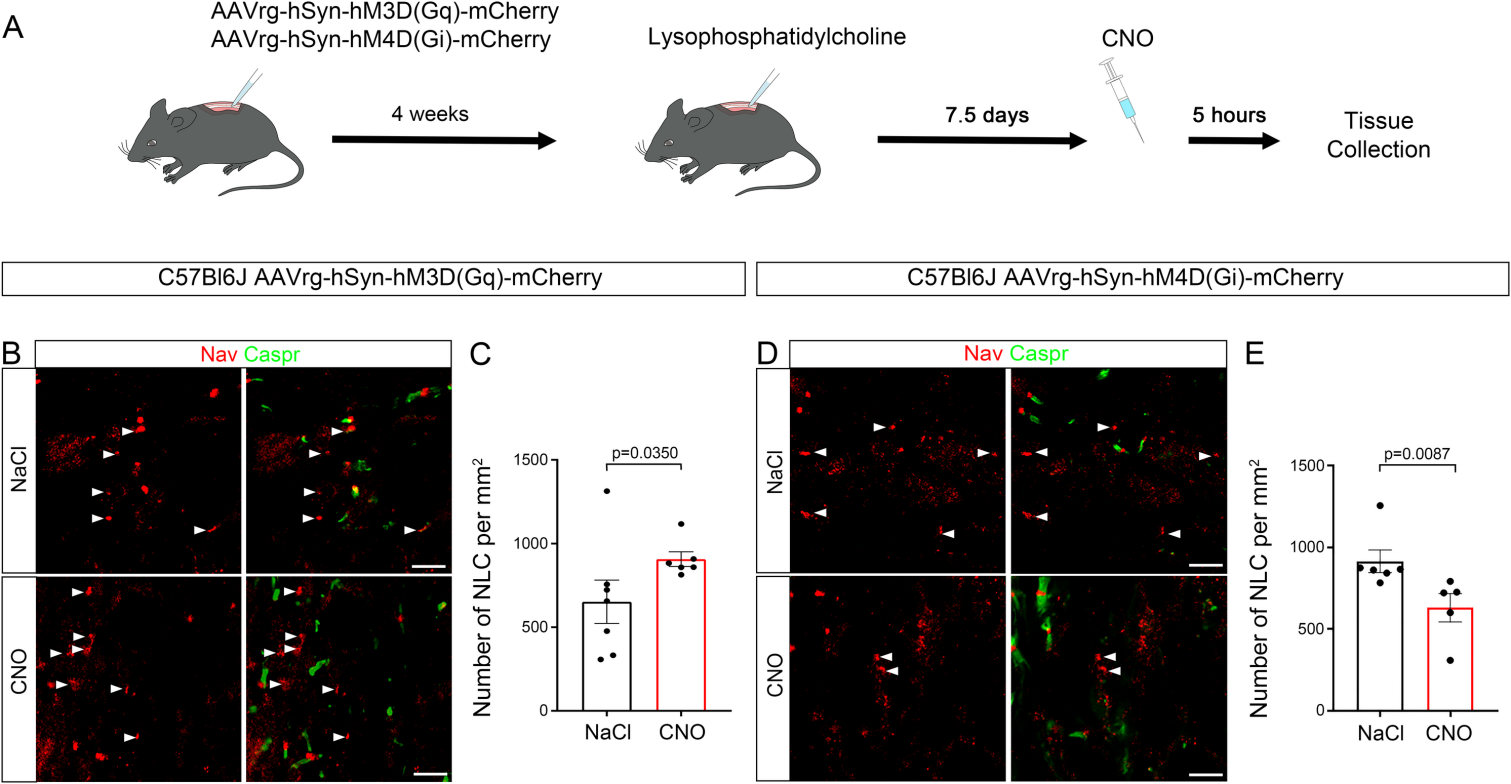
Neuronal activity increases node‐like cluster formation prior to remyelination in mouse spinal cord. (A) For the DREADDs approach, mice were transduced with a retrograde virus AAV‐hSyn‐hM3D(Gq)‐mCherry or AAV‐hSyn‐hM4D(Gi)‐mCherry in the dorsal funiculus of the spinal cord. A focal demyelination was then induced 4 weeks after the viral transduction, by injection of lysophosphatidylcholine in the same tractus to induce demyelination. Following the peak of demyelination, N‐Clozapine Oxide (CNO) or NaCl (Control) was injected intraperitoneally at 7.5 DPI, 5 h before perfusion of the animals. (B) Representative images of nodal clusters within the lesion of mice expressing hM3D(Gq)‐mCherry. (C) Quantification of the number of node‐like clusters in the lesion following CNO or NaCl injection in mice expressing hM3D(Gq)‐mCherry. (D) Representative images of nodal structures within the spinal cord lesion of mice expressing hM4D(Gi)‐mCherry. (E) Quantification of the number of node‐like clusters in the lesion of mice expressing hM4D(Gi)‐mCherry. (B, D) White arrowheads show node‐like clusters (Nav, red) without paranodal staining surrounding them (Caspr, green) in CNO and NaCl condition. (C, E) Each point corresponds to one animal, (C) *n* = 7 for NaCl and *n* = 6 for CNO, Mann–Whitney test. (E) *n* = 6 for NaCl and *n* = 5 for CNO, Mann–Whitney test. (B, E) Scale Bar: 10 μm.

We found that node‐like clusters are formed along the dorsal tract of the mouse spinal cord during remyelination in vivo (Figure 6). We next used an in vivo demyelinating mouse model, corresponding to a focal injection of LPC in the dorsal funiculus of the adult mouse spinal cord (as described in Ronzano et al. 2021), to investigate the effect of neuronal activity on node‐like clusters in vivo. To modulate neuronal activity, we used retrograde AAV inducing the expression of hM3D(Gq)‐mCherry or hM4D(Gi)‐mCherry and targeted the supraspinal neurons sending descending projections to the spinal cord via the dorsal tracts (Wang et al. 2018). We activated the DREADD receptors 7.5 days post LPC injection (after the peak of demyelination) by CNO intraperitoneal injection, with control animals receiving NaCl 0.9% in place. We validated the expression and activation of the DREADD receptors in vivo by assessing cFos expression (immediate‐early gene which expression is activated by neuronal activity; Hunt et al. 1987) in mCherry expressing neurons (corticospinal neurons; Figure S8A–C). We observed a significant increase of cFos expression following hM3D(Gq)‐mCherry activation, while the number of cFos positive cells was reduced following hM4D(Gi)‐mCherry activation (Figure S8D,E respectively). We then quantified the density of node‐like clusters within the lesion at 7.5 days post LPC injection, following CNO treatment and in control condition.

Following neuronal activity induction, in the case of hM3D(Gq) receptor, we observed a 1.4‐fold increase of node‐like cluster density in CNO treated vs. control animals (Figure 6B,C). In a second batch of experiments, we inhibited neuronal activity at a similar timepoint post LPC injection by activation of hM4D(Gi) receptor, which led to a 31% decrease of node‐like cluster density in CNO treated animals compared to controls (Figure 6D,E).

These results show that neuronal activity also promotes node‐like cluster assembly in remyelination in vivo.

## 3 Discussion

In this work, we show using in vitro to in vivo models that neuronal activity modulates node‐like cluster assembly prior to myelination in various neuronal subtypes and present evidence of a similar regulatory process during remyelination, suggesting this could be a general feature of node‐like cluster assembly.

### 3.1 Neuronal Activity and Glutamatergic Excitatory Signals Can Modulate Node‐Like Cluster Formation Through Neuronal Intrinsic Mechanisms

Neuronal activity and glutamatergic inputs are known to modulate the oligodendroglial lineage (De Biase et al. 2010; Káradóttir et al. 2005; Spitzer et al. 2016; Ziskin et al. 2007), which promotes node‐like clustering through secreted factors (for review, Lubetzki, Sol‐Foulon, and Desmazières 2020). Astrocytes can further express NMDA receptors (Skowrońska et al. 2019). To address the possibility of an indirect effect of neuronal activity on node‐like cluster assembly through glial cell modulation, we used purified hippocampal neuron cultures supplemented with oligodendrocyte conditioned medium (OCM) in parallel with mixed hippocampal cultures to study the effect of glutamatergic signaling inhibition. Using this approach, we still observed a modulation of node‐like cluster formation while inhibiting glutamatergic inputs, confirming a cell‐autonomous modulation of node‐like clusters in GABAergic neurons. Thus, neuronal activity can regulate node‐like cluster assembly through a direct modulation of intrinsic neuronal properties, though an additional role of glial cells in response to glutamatergic inputs cannot be excluded. Kaplan and colleagues had previously observed that modulating activity and glutamatergic inputs pharmacologically in retinal ganglion cells cultured with OCM had no significant effects on cluster spacing in this cell population (Kaplan et al. 2001). The results regarding clustering per se were somehow contradictory, though a tendency to increased clustering was observed following glutamatergic agonists treatment (Kaplan et al. 2001).

We observed that NMDA (but not AMPA/Kainate receptor agonists) can partially counteract the inhibitory effect of TTX on node‐like cluster formation in purified neuron cultures treated with OCM. This suggests that the positive effect of glutamatergic inputs on node‐like clustering in hippocampal GABAergic neurons is prominently mediated by NMDA receptors and associated downstream pathways.

In addition, reinforcing neuronal activity was not sufficient to induce node‐like cluster formation in hippocampal glutamatergic neurons, which do not assemble these clusters under normal conditions. This further suggests that neuronal activity modulates intrinsic properties specific to the neuronal subpopulations assembling node‐like clusters. Our data further suggest that neuronal activity modulation could lead to variations in node‐like cluster spacing in hippocampal GABAergic neurons, without significantly affecting nodal length, but the possibility that neuronal activity can modulate the characteristics of node‐like clusters themselves (such as density and subtypes of channels) and whether this could further impact conduction velocity, as observed for mature nodes (Arancibia‐Carcamo et al. 2017; Cullen et al. 2021; Ford et al. 2015), remains to be addressed.

### 3.2 Glutamatergic Signaling Modulates the Expression of Node‐Like Cluster Proteins

We further investigated which neuronal mechanisms could drive node‐like cluster assembly modulation by neuronal activity and glutamatergic signaling, and focused on nodal component expression modulation and axonal transport of these proteins, two mechanisms we previously showed to be involved in node‐like cluster formation in vitro (Kaplan et al. 2001; Thetiot et al. 2020). Here, we demonstrated that the inhibition of glutamatergic transmission leads to a decreased expression of various nodal proteins. Though neuronal activity is described to modulate axonal trafficking (Chen and Sheng 2013; Ohno et al. 2011), we did not observe alteration of nodal marker axonal transport following the inhibition of excitatory glutamatergic neurotransmission.

Neuronal activity and NMDA receptors are both known to be implicated in gene expression modulation (Flavell and Greenberg 2008; Hrvatin et al. 2018; P. Hu et al. 2017; Lacar et al. 2016; Yap and Greenberg 2018), and glutamatergic excitatory inputs were shown to participate in GABAergic interneurons post‐natal maturation, at least in part through transcriptional regulation (for review: Lim et al. 2018). Moreover, we previously reported that in hippocampal cultures, GABAergic neurons with node‐like clusters receive higher frequencies of excitatory synaptic events. They further differ in their transcriptome from GABAergic neurons without clusters and pyramidal cells, and notably upregulate *Scn1a* expression (Mazuir et al. 2021). Furthermore, Mahadevan and colleagues have recently observed a reduced expression of *Scn1a* and *Scn1b* in GABAergic neurons following a loss of function of the NMDAR subunit GRIN1 (Mahadevan et al. 2021).

These results are consistent with our findings. We show that the inhibition of glutamatergic transmission leads to a decreased expression of *Scn1a*, encoding the voltage‐gated sodium channel isoform Nav1.1, and *Scn1b*, encoding β1Nav, without affecting *Scn2a* expression, encoding Nav1.2.

This upregulation might participate in the very high density of Nav channels observed in fast‐spiking GABAergic neurons, which may ensure fast signaling and compensate for the unfavorable morphological properties of their axons (H. Hu et al. 2014; H. Hu and Jonas 2014). Nav1.1 expression is a common feature of all the neuronal subtypes described to form node‐like clusters (Duflocq et al. 2008; Freeman et al. 2015; Kaplan et al. 1997, 2001; Van Wart et al. 2007), and in the hippocampus, its axonal expression is restricted to GABAergic cells, the only node‐like forming neurons. In the present study, we completed these results by showing the role of glutamatergic inputs in the upregulation of Nav1.1 expression. We further showed that inhibiting Nav1.1 leads to a strong reduction of node‐like cluster formation in GABAergic neurons in vitro, although other Nav channels are still present as shown by the persistence of PanNav staining and *Scn2a* expression. Furthermore, the fact that the reinforcement of *Scn2a* expression following *Scn1a* knock‐down does not allow the rescue of node‐like clustering, and the inhibition of *Scn2a* expression does not affect node‐like cluster formation further supports the essential role of Nav1.1 in node‐like cluster formation. Altogether, this strongly supports that Nav1.1 regulation is part of the mechanism through which neuronal activity regulates node‐like cluster assembly.

We have previously shown that node‐like cluster formation and restriction stabilization is a two‐step mechanism, with first a targeting of αNav/β2Nav/AnkG as loose clusters, followed by Nfasc186/NrCAM/β1Nav recruitment, which is associated with cluster restriction and stabilization (Thetiot et al. 2020). Neuronal activity, by modulating Nav1.1 and β1Nav expression, may contribute to support both these steps.

This cell‐autonomous mechanism may further synergize with the secretion of glial factors known to participate in node‐like clustering (Dubessy et al. 2019; Elbaz et al. 2024), leading to a fine tuning of this process.

### 3.3 An Indirect Modulation of Myelination and Neuronal Networks by Neuronal Activity Through Node‐Like Cluster Formation in Development, Plasticity and Repair

Neuronal activity‐dependent node‐like cluster assembly could participate in various aspects of developmental processes, including neuron physiology and neuronal network establishment. Glutamatergic signaling alteration in GABAergic neurons has been associated with neurodevelopmental psychiatric disorders, but whether node‐like clusters are affected in these diseases has not yet been investigated. In addition, node‐like clustering characterizes mostly neurons that are programmed to be subsequently myelinated (Lubetzki, Sol‐Foulon, and Desmazières 2020), and our results suggest that node‐like cluster assembly can be an intermediate step of activity‐dependent myelination.

The formation of node‐like clusters along axons prior to myelination has been shown to participate in mature node formation (Malavasi et al. 2021; Thetiot et al. 2020; Vagionitis et al. 2022), some node‐like clusters predefining mature nodes being thus potentially landmarks for future myelination pattern. Furthermore, these clusters may regulate myelination itself by their role in conduction modulation and myelin deposition guidance (Freeman et al. 2015; Thetiot et al. 2020). For this later process, local vesicular release may be at play, as it was shown to regulate myelin sheath stabilization and growth (Almeida et al. 2021; Hines et al. 2015; Koudelka et al. 2016; Mensch et al. 2015), as well as promote MBP local synthesis in oligodendrocytes at close contact of release site (Wake et al. 2015).

This cascade of events may affect the timing of axonal transmission, which is critical for neuronal network synchronization and myelination (Arancibia‐Carcamo et al. 2017; Freeman et al. 2016; Monje 2018; Seidl 2014). It would thus be of particular interest to investigate whether some non‐myelinated or partially myelinated neurons can form node‐like clusters in response to increased neuronal activity in adult, therefore participating in plasticity through refinement of axonal conduction and adaptive myelination (Bacmeister et al. 2022; Gibson et al. 2014; McKenzie et al. 2014).

In the present work, we show that node‐like cluster assembly is also promoted by neuronal activity prior to remyelination. In demyelinating pathologies such as multiple sclerosis, remyelination exists but is only partial and its failure may lead to neuronal loss. Node‐like clusters have been previously described in remyelinating MS plaques and we observe them in MS rodent models. The mechanism and function of node‐like cluster formation prior to remyelination remain elusive, and whether they facilitate axonal conduction along demyelinated axons and/or promote myelin redeposition in an unfavorable environment has not yet been disentangled. In the present work, we show that node‐like cluster assembly is also promoted by neuronal activity prior to remyelination, along axons whose remyelination can be modulated by activity (Perrot et al. 2025).

Extrapolating what has been suggested for developmental myelination (Vagionitis et al. 2022), it can be hypothesized that node‐like clusters may participate in defining the pattern of remyelination along axons following demyelination (Auer et al. 2018; Orthmann‐Murphy et al. 2020; Snaidero et al. 2020). Of note, following cuprizone‐induced demyelination, the scaffolding nodal protein βIV Spectrin can remain clustered (Orthmann‐Murphy et al. 2020), possibly serving as a landmark for remyelination pattern. In contrast, the myelination pattern is not restored in some cortical areas, though the global amount of myelin is (Bacmeister et al. 2022). Whether this is a defect in remyelination or an adaptation to remyelinate the most active neurons following axonal injury in cortical areas is not known. Assessing the presence and potential role of activity‐dependent node‐like cluster formation in this context could give further insights on this process.

In conclusion, neuronal activity appears to support the assembly of node‐like clusters, which in turn modulate axonal conduction and guide myelin deposition initiation. This could participate in the adequate establishment of neuronal networks during development, as well as myelination regulation and patterning during developmental myelination, plasticity, and remyelination.

## 4 Materials and Methods

### 4.1 Animals

The care and use of mice conformed to institutional policies and guidelines (UPMC, INSERM, French and European Community Council Directive 86/609/EEC). The following mouse strains were used in this study: C57bl6/J (Janvier Labs), L7‐ChR2‐eYFP (gift from Prof *C. Lena* , IBENS, PSL University, Paris, France; Chaumont et al. 2013), transferred into a C57bl6/J genetic background and used after a minimum of six generations. Wistar pregnant rat females were purchased from Janvier Labs.

### 4.2 Hippocampal Mixed Culture

Mixed hippocampal cultures (containing neurons and glial cells) were made from rat embryos as previously described (Freeman et al. 2015). Briefly, pooled hippocampi of E18 rat embryos were dissociated enzymatically by trypsin treatment (0.1%; Worthington) with DNase (50 μg/mL, Worthington) for 25 min at 37°C. Following trypsin neutralization, cells were mechanically dissociated, centrifuged at 400×*g* for 5 min, resuspended, and seeded on polyethylenimine (Sigma) precoated glass coverslips at a density of 5.0 × 10^4^ cells per well in 24 well plates (surface of 35mm^2^ per well, TPP) or per 35 mm glass‐bottom dishes (81,158; Ibidi, BioValley). Cultures were maintained for 24 h in a 1:1 mixture of DMEM Glutamax (31966047, ThermoFisher Scientific) with 10% fetal calf serum (10270106, ThermoFisher Scientific), penicillin–streptomycin (100 IU/mL, 1514022, ThermoFisher Scientific), and neuron culture medium (NCM). Culture medium was replaced by a 1:2 mixture of Bottenstein–Sato (BS) medium with PDGF‐A (0.5%, Peprotech) and NCM, and then, half of the medium was removed every 3 days and replaced by NCM. The NCM contained neurobasal medium (21,103,049; ThermoFisher Scientific) supplemented with 0.5 mM L‐glutamine (25,030,024, ThermoFisher Scientific), B27 (17,504,044, ThermoFisher Scientific), and penicillin–streptomycin (100 IU/mL each). The BS medium was made of DMEM Glutamax supplemented with transferrin (100 μg/mL), albumin (100 μg/mL), insulin (5 μg/mL), progesterone (60 ng/mL), putrescine (16 μg/mL), sodium selenite (40 ng/mL), T3 (40 ng/mL), and T4 (40 ng/mL), all from Sigma.

### 4.3 Organotypic Cultures of Mouse Cerebellar Slices

Cerebellar slice cultures were made as previously described (Birgbauer et al. 2004; Thetiot et al. 2019). Briefly, P8‐10 mouse cerebella were dissected in ice cold Gey's balanced salt solution (G9779, Sigma) complemented with 4.5 mg/mL D‐Glucose (G8769‐100ML, Sigma) and 1× penicillin–streptomycin (100 IU/mL, ThermoFisher Scientific) before being cut into 250 μm parasagittal slices using a McIlwain tissue chopper and placed on Millicell membrane (3–4 slices each per animal, 0.4 μm membranes, Merck Millipore) in 50% BME (41010026, ThermoFisher Scientific), 25% Hanks' Balanced Salt Solution (14185‐045, ThermoFisher Scientific), and 25% heat‐inactivated horse serum (26050088, ThermoFisher Scientific) medium, supplemented with GlutaMax (2 mM, 35050038, ThermoFisher Scientific), penicillin–streptomycin (100 IU/mL, ThermoFisher Scientific), and D‐Glucose (4.5 mg/mL; Sigma). The slices from a given animal were divided on two membranes, one of the membranes being used as control, the second being treated to minimize the potential variability and do paired experiments. Cultures were maintained at 37°C under 5% CO_2_ and medium changed every 2–3 days. The experiments were analyzed at 3 days in vitro (DIV, onset of myelination), 4 DIV (myelination), and 10 DIV (onset of remyelination).

### 4.4 Viral Transduction of In Vitro and Ex Vivo Cultures

The adenoviruses used to express hM3Dq or hM4Di fused to the reporter mCherry under the control of the human Synapsin promoter, AAV8‐hSyn‐hM3D(Gq)‐mCherry and AAV8‐hSyn‐hM4D(Gi)‐mCherry are available commercially (gift from Bryan Roth, Addgene plasmids #50474‐AAV8 and #50475‐AAV8 respectively) and drove the expression of DREADDs receptors in neurons. In hippocampal mixed cultures, the transduction was performed at 6 DIV, by adding the virus at a final concentration of ~10^9^ VP/μL, when renewing half of the medium. Regarding organotypic slice cultures, the transduction was performed immediately following slice preparation by addition of the AAV solution directly onto the slices placed on milicell membranes (1 μL/slice at a final concentration of 1 × 10^11^ and 5 × 10^11^ VP/μL for AAV8‐hSyn‐hM3D(Gq)‐mCherry and AAV8‐hSyn‐hM4D(Gi)‐mCherry respectively).

### 4.5 Hippocampal Neuron Culture and Organotypic Culture Treatments

#### 4.5.1 Pharmacological Treatment of Hippocampal Neuron Cultures and Cerebellar Slices

To activate the DREADDs receptor hM3Dq or hM4Di, the culture medium was renewed with a culture medium containing 0.5 μM N‐Clozapine (CNO, 0.5 μM, Cayman chemical, 16882) diluted in DMSO (700001, Cayman Chemical) or a culture medium with an equivalent volume of DMSO (1 μL in 1 mL of medium) in control condition. For hippocampal mixed cultures, CNO treatment started at 8 DIV and was maintained until fixation of the cell culture (DIV17 for hM4G(Gi)‐mCherry and DIV 14 for hM3D(Gq)‐mCherry). The cerebellar slices were treated with 0.5 μM CNO or DMSO (control condition) at 3 DIV (myelination) or 10 DIV (remyelination). The slices were treated 6 h with CNO to activate hM3D(Gq)‐mCherry and fixed 1 h after the end of the treatment to assess the effect on node‐like cluster formation. To study the impact of neuronal activity on myelination, the slices were treated with CNO or DMSO as previously done, but were fixed the following day.

To study the effect of neuronal activity and glutamatergic inputs on node‐like cluster assembly in hippocampal neuron cultures, at 8 DIV half of the culture medium was renewed with an equivalent volume of NCM containing the different drugs at 2×, either tetrodotoxin (TTX, Sigma T2265‐25G; final concentration 0,1 μM), or glutamatergic receptor inhibitors, kynurenic acid (KYN; final concentration 1 mM, Sigma), NBQX (final concentration 10 μM, AbCAM) or APV (final concentration 100 μM, AbCAM). To further study the effect of glutamatergic inputs « per se » on GABAergic neurons, hippocampal neuron cultures supplemented with OCM were treated at 8 DIV with TTX 0.1 μM final and the glutamatergic agonist NMDA (20 μM final, Sigma) or quisqualic acid (AcQ, AMPA and kainate agonist, 1 μM final, Sigma). After addition of the treatments to the cultures, half of the medium was renewed every 3 days with NCM with drug 1× until fixation at 17 DIV. To study the effect of glutamatergic inputs on node‐like‐cluster assembly in Purkinje cells prior myelination, kynurenic acid (KYN) was added to the culture medium directly following slice generation to a final concentration of 1 mM, and the slices were fixed at 3 DIV.

#### 4.5.2 Demyelination of Organotypic Cerebellar Slices

To induce demyelination, for each animal, the myelinated slices were incubated 16 h at 6 DIV in 0.5 mg/mL l‐α‐Lysophosphatidylcholine (LPC, 830071P, Avanti, Merck) added to fresh culture medium.

### 4.6 Optogenetics Stimulation of Organotypic Cerebellar Slices

Optogenetics stimulations were performed on the organotypic cerebellar slices with a custom‐designed set‐up that allows stimulation with light at 470 nm directly on 6‐well culture plates (Desmazieres, Ronzano and Marty). Each well is illuminated by a LED (SZ‐05‐H, Luxeonstar), each LED being individually calibrated to allow a stimulation at a power of 1.5 mW/mm^2^ on the slices. The pattern of stimulation was controlled with an Arduino (A000067, Arduino Mega 2560, Arduino, ref.782‐A000067, Mouser Electronic). To stimulate Purkinje cells, we used 10 ms long pulses at 10 Hz for 6 h applied at 3 DIV or 10 DIV and fixed the slices 1 h after the end of the stimulation to study node‐like cluster formation prior to myelination or remyelination respectively. To study the impact of neuronal activity on myelination, the slices were stimulated as usual, but fixed the following day (4DIV). To avoid cytotoxicity, prior to stimulation, the membranes were transferred in a medium free of phenol‐red consisting of: 75% DMEM (11,880,028, Gibco, ThermoFisher Scientific), 20% 1× HBSS (14185‐045, Gibco, ThermoFisher Scientific) supplemented with HCO_3_^−^ (0.075 g/L final; 25080060, Gibco, ThermoFisher Scientific), 5% heat‐inactivated horse serum (ThermoFisher Scientific), HEPES Buffer (10 mM final; 15630056, Gibco, ThermoFisher Scientific), D‐Glucose (4.5 g/L final; G8769‐100ML, Sigma), GlutaMax (2 mM final; 35050038, ThermoFisher Scientific) and penicillin–streptomycin (100 IU/mL each; ThermoFisher Scientific).

### 4.7 In Vivo Intraspinal Injections

#### 4.7.1 Viral Injection

Following an intraperitoneal injection of buprenorphine (0.1 mg/mL, Buprecare, Med'Vet) done 15 min before surgery to prevent pain, C57Bl6J females (8 weeks old, 18‐22 g) were anesthetized with isofluorane (3% induction, 1,5% maintenance, Vetfluran). The anesthetized animals were then placed onto a spinal stereotaxic frame and an incision was made between the thoracic and lumbar vertebrae to access the dorsal funiculus of the spinal cord. 1 μL of AAVrg‐hSyn‐hM3D(G_q_)‐mCherry (Addgene, #50474‐AAVrg, 2.5 × 10^13^ GC/mL) or AAVrg‐hSyn‐hM4D(G_i_)‐mCherry (Addgene, #50475‐AAVrg, 2.4 × 10^13^ GC/mL) were then injected intraspinally with a glass capillary (Wang et al. 2018). Following surgery, the mice were stitched and placed until recovery into a warming chamber (Vet tech solution LTD, HE011).

#### 4.7.2 Focal Spinal Cord Demyelination

The focal demyelination was induced 4 weeks post viral injection. The initial steps of the surgery protocol are similar to the viral injection described above. An incision is made between the thoracic and lumbar vertebrae to access the dorsal funiculus of the spine, and an intraspinal injection of 1 μL of Lysophosphatidylcholine diluted into NaCl 9‰ (LPC, 10 mg/mL; Sigma‐Aldrich L4129) is made with a glass capillary. Following surgery, the mice were stitched and placed until recovery into a warming chamber (Vet tech solution LTD, HE011). The peak of demyelination is reached around 7 days post injection, and remyelination starts from 10 days post injection.

### 4.8 DREADDs Receptors Activation In Vivo

Clozapine N‐Oxide (CNO, Cayman Chemical Company) was diluted into NaCl 9‰ to a final concentration of 1 mg/mL and the solution was injected intraperitoneally (5 mg/kg, two injections with a 2‐h interval). The mice were then euthanized with Euthasol (Centravet) and transcardially perfused with 2% PFA (Electron Microscopy Services, ThermoFisher Scientific) 5 h after the initial CNO injection, at 7.5 days post LPC injection.

### 4.9 Analysis

#### 4.9.1 In Vitro Axonal Transport Analysis

For the analysis of axonal transport live imaging in vitro, kymographs (representing the trajectory of the fluorescent puncta over time) were generated with the ImageJ software, using the KymoToolBox plugin (kindly provided by Dr. F. Cordelière, University of Bordeaux, France). For analysis, individual trajectories were manually traced. For all kymographs, moving puncta [anterograde (toward the axon terminal), retrograde (toward the cell body), or bidirectional] were defined as previously described (Thetiot et al. 2020). We calculated the mean number of puncta per 100 μm in each condition and the distribution of anterograde, retrograde, bidirectional, and stationary puncta. Group analysis of the trajectories was performed using a homemade Excel macro designed by Dr. J. Tailleur (University of Paris Diderot, France and MIT, Cambridge, USA). The data presented correspond to the analysis of 17–33 neurons, from three to four experiments. Data are presented as the mean ± standard error of the mean (SEM) of the experiments.

#### 4.9.2 Study of Nodal Marker Expression in GABAergic Neurons In Vitro

GABAergic neurons were colabelled for GAD67, PanNav and a nodal marker of interest (β1Nav, β2Nav, Nfasc, Nav1.1, Kv3.1b or Kv7.2) at 17 DIV, following KYN treatment or in control condition. The number of GAD+ neurons with node‐like clusters (defined by PanNav expression) was determined using an upright Axio imager microscope (Zeiss) with a 40× oil immersion objective with a numerical aperture of 1.3. A Nav cluster was defined by a length between 1 and 8 μm and a neuron was counted as positive for node‐like clusters when at least 2 Nav clusters were observed along its axon (Freeman et al. 2015). The expression of the tested nodal marker of interest was assessed in NLC+ and NLC‐ GAD+ populations. A total of about 250 to 600 GAD+ neurons were counted per condition, from three to four independent experiments (biological replicates). Results are expressed as mean ± SEM of the experiments.

#### 4.9.3 Quantification of Node‐Like Clusters

For hippocampal mixed cultures, miRNA transfected or DREADDS expressing GABAergic neurons were labeled for GAD67 and GFP or mCherry respectively. Node‐like clustering was quantified at 14 DIV (initiation of node‐like clustering) when enhancing activity and at 17 DIV (when a higher density of node‐like clusters is observed) when inhibiting activity to maximize the potential variations of node‐like clustering due to neuronal activity modulation. The number of GAD67+/GFP+ or mCherry+ neurons with node‐like clusters was quantified as described above. On average, about 60–200 GAD+ or GAD+/GFP+ or mCherry+ neurons were counted per experiment per condition, and at least three independent experiments were performed (biological replicates). Results are expressed as mean ± SEM.

For the cerebellar slice cultures at 3–4 DIV (myelinating slices), 8 DIV (demyelinated slices), 10–11 DIV (remyelinating slices) and for the cerebellum sections at P10, the density of node‐like clusters (defined as nodal marker cluster not flanked by paranodal structure(s) or myelin) was quantified on one optical section in the middle of the image stack. The rest of the stack was used to make sure that the structures corresponded to a node‐like cluster and not to an axon initial segment orthogonal to the imaging plane. The quantification was performed on five different fields of view per animal, and the number of node‐like clusters per area unit was averaged to give the mean value for one animal. The analysis was performed in at least five animals per condition from at least two different independent litters.

Following neuronal activity stimulation with both DREADDs, optogenetics, and KYN treatment, the proportion of Purkinje cells with node‐like clusters and the number of node‐like clusters was assessed along the first 130 μm of the axon starting from the distal end of the axon initial segment. When the axon had a branch point along these 130 μm, the analysis was made along the main branch going toward the white matter tract. The analysis was reduced to this part of the axon to restrain the quantification to the area where myelination was ongoing. Indeed, during cerebellar development, the myelination proceeds in an ascending manner along Purkinje axons from the white matter tract toward the soma (Gianola et al. 2003). The proportion of Purkinje cells with node‐like clusters was quantified on four to five different fields of view to obtain, on average, 32 (range: 16–48) mCherry+ or YFP+ Purkinje cells per condition per animal for myelinating condition and, on average, 31 (range: 21–38) for remyelination.

To analyze node‐like cluster assembly in vivo during remyelination, the number of node‐like clusters, identified as isolated Nav clusters (i.e., without Caspr staining surrounding the cluster) was quantified on a central image of the image stack, the surrounding images further being used to exclude the possibility of the structures being heminodes or mature nodes. For each image, the density of node‐like clusters was calculated as the number of clusters per image area, brought back to the number of clusters per mm^2^ and the mean density was calculated using the five images acquired per animal. The results are presented as the mean number of node‐like clusters per area per animal (*n* = 7 animals hM3/NaCl, *n* = 6 animals hM3/CNO, *n* = 6 animals hM4/NaCl and *n* = 5 animals hM4/CNO).

#### 4.9.4 Statistics

All statistical analysis and data visualization were carried out using Prism (GraphPad, version 7) and R software version 3.6.2. For all the experiments, the number of biological replicates and statistical tests applied are reported in the text or in the figure legends, as well as in the summary Table. Graphs and data in the text are reported as the mean ± SEM; each biological replicate is individually plotted. The level of statistical significance was set at *p* < 0.05 for all tests.

When the sample size was *n* ≥ 6, we assessed for the normality of the distribution using a Shapiro–Wilk test and used accordingly parametric tests when the distribution was not significantly different than a normal distribution, and non‐parametric tests were applied otherwise. When the sample size was *n* < 6, parametric tests were used assuming the normality of the distributions since in these cases each individual sample represented a large number of repeated measures.

For the in vitro studies, Student's *t*‐test followed by correction using FDR method when required or One‐ or Two‐way ANOVA followed by multiple comparison tests were used when appropriate. For the effect of neuronal activity on node‐like cluster assembly ex vivo, the design involved a pairing; therefore, groups were compared using two‐sided paired tests. For the quantification of node‐like clusters density in vivo and ex vivo, two‐sided unpaired tests were used. All the statistical results are summarized in the Table S1.

## Author Contributions

Anne Desmazières, Nathalie Sol‐Foulon, Rémi Ronzano, Clément Perrot designed the research. Rémi Ronzano, Clément Perrot, Elisa Mazuir, Melina Thetiot, Marie‐Stéphane Aigrot, Paul Stheneur, Nathalie Sol‐Foulon, and Anne Desmazières did the experiments and analysis. François‐Xavier Lejeune participated in statistical analysis. Anne Desmazières, Nathalie Sol‐Foulon, Rémi Ronzano, Clément Perrot, Bruno Stankoff, and Catherine Lubetzki wrote the publication.

## Funding

This work was funded by Fondation pour l'Aide à la Recherche sur la Sclérose en Plaques (France Sclérose en plaques) grants to AD and NSF, BBT‐IHU grant to AD and Institut du Cerveau et de la Moelle Epinière, Institut National de la Santé et de la Recherche Médicale and Biogen support.

## Conflicts of Interest

The authors declare no conflicts of interest.

## Supporting information


**Figure S1:** The inhibition of glutamatergic receptors affects the expression of some nodal protein, but does not impact nodal marker axonal transport. The inhibition of glutamatergic inputs in mixed hippocampal cultures by the addition of a glutamatergic antagonist (kynurenic acid, KYN, 1 mM) affects the expression in GABAergic neurons (GAD+, white) of Kv3.1b (A, E), while Kv7.2 (B, F), Nfasc (C, G) and β2Nav (D, H) are still expressed in the vast majority of GABAergic neurons. Of note, at 17 DIV, in control condition, Kv7.2 was restricted to the axon initial segment and node‐like clusters when present. Kv3.1b, though strongly expressed in some GAD+ neurons, was not restricted to, nor enriched at axonal domains. (E‐H) Percentages of GAD+ neurons expressing the nodal marker of interest (percentage of cells with node‐like clusters in dark and of cells without clusters in white) in control condition or following treatment by KYN. Unpaired t‐test. Scale bars: 20 μm. (I‐K) Expression (+ or ‐) of Nav1.1 (I), β1Nav (J) and Kv3.1b (K), in GABAergic neurons with or without clusters (NLC + or—respectively) in control condition (black bars) or following kynurenic acid treatment (red bars). (L) Representative kymographs illustrating β1Nav‐ and β2Nav‐mCherry axonal transport at 17 DIV following kynurenic acid treatment (KYN) compared to control condition. Anterograde transport from left to right. (M‐P) Quantification of the total number of mCherry+ puncta per 100 μm (M, O) and mCherry+ puncta category distribution (N, P) for β1Nav‐ and β2Nav‐mCherry following KYN (red bars) treatment compared to control (black bars). FO: forward, BA: backward and BI: bidirectional moving puncta. ST: stationary puncta. The histograms show the means ± SEM. (M‐O) Unpaired t test, (N, P) Two‐way ANOVA followed by Tukey's multiple comparisons test. β1Nav‐mCherry: *n* = 4 experiments; β2Nav‐mCherry: *n* = 3 experiments. (L) Scale bar: 5 s.

**Appendix S1:** Supporting Information.

**Figure S2:** Selective knockdown of Scn1a and Scn2a expression using miRNA‐based silencing in transfected GABAergic neurons. (A) RNAscope combined with immunostaining in mixed hippocampal cultures reveals a strong reduction of Nav1.1 encoding mRNA expression (Scn1a, red), in transfected GABAergic neurons (GAD+, white; emGFP+, green) expressing miR 254 or miR 947, both targeting Scn1a mRNA, compared to those expressing miR Control or miR 215 (targeting Scn2a RNA), with a strong decrease in red puncta observed in the cell body of GAD+/emGFP+ neurons. (B) RNAscope detection of Nav1.2 encoding mRNA (Scn2a, red) shows a strong reduction of Scn2a mRNA in transfected GAD+/emGFP+ GABAergic neurons expressing miR 215 compared to miR Control, confirming its efficacy in silencing Scn2a expression. We further observed that GABAergic neurons expressing miR 254 or miR 947 (targeting Scn1a) exhibit a strong increase in Scn2a mRNA signal, as observed with a strong reinforcement of red puncta in their cell body, suggesting a compensatory upregulation of *Scn2A* expression in response to *Scn1a* kock‐down. Scale bars: 10 μm.

**Figure S3:** glia70138‐sup‐0004‐FigureS3.tif. *Scn2a* downregulation does not alter node‐like cluster formation. At DIV17, node‐like cluster assembly (Nav, in red; white arrowheads) is observed in GABAergic neurons (GAD, in white) expressing control miRNA (green, upper lane) and *Scn2a‐*targetting miRNA 215 (green, lower lane). Scale bars: 30 μm.

**Figure S4:** Node‐like clusters are formed prior to myelination and remyelination along Purkinje cells axons. (A) Immunohistostainings of a cerebellar cultured slice at 4 DIV showing node‐like clusters (Nav in red, filled arrowheads) without paranodal clustering (gray, Caspr), distributed in regions with ongoing myelination (PLP, in green). The diffused Caspr signal allows to follow unmyelinated Purkinje axons. (B) Example of an isolated node‐like cluster (Nav in red, filled arrowhead) surrounded by two heminodes (contour arrowheads) along the same axons. (C) Orthogonal projections showing isolated node‐like clusters (Nav in red, filled arrowhead) without paranodal clustering (gray, Caspr) in a remyelinating (PLP, in green) cerebellar slice at 11 DIV. (D) Orthogonal projection of a sagittal section of the cerebellum at P10 showing Purkinje cells axons (Calbindin, in gray), with node‐like clusters (Nav in red, filled arrowhead) along unmyelinated part of their axons (myelin stained by PLP, green). (E) Orthogonal projection showing at a higher magnification two node‐like clusters (Nav in red, filled arrowhead) along an unmyelinated portion of the same axon and heminodes (contour arrowhead) at the extremity of the myelin sheathes. (F) Quantifications of node‐like clusters in the cerebellum in vivo at P10, ex vivo at 3–4 DIV and ex vivo in remyelinating area at 11 DIV show similar densities of node‐like structures in the regions with ongoing myelin deposition. Each value individually plotted corresponds to 1 animal, in vivo P10 and ex vivo 11 DIV: *n* = 4 animals, ex vivo 4 DIV: *n* = 5 animals. One‐way ANOVA. **(G‐H)** Cultured cerebellar slices at 8 DIV (peak of demyelination, G) and 10 DIV (onset of remyelination, H), showing nodal structures clusters (Nav, red), paranodal regions (Caspr, gray), and myelin (PLP, green). Node‐like clusters are indicated by filled arrowheads. (I) Quantification of node‐like clusters in demyelinated and remyelinating conditions. *n* = 4 animals in each condition. Paired *t*‐test. Scale bars: (A, C) 20 μm, (B,D) 5 μm, (D) 10 μm, (G,H) 15 μm.

**Figure S5:** Modulations of the firing activity of Purkinje cells in organotypic cultures of cerebellar slices by DREADD and optogenetic stimulations. (A) Example of a hM3D(Gq)‐mCherry transduced Purkinje cell (filled white arrowhead) recorded in loose cell‐attached voltage clamp. (B) Representative example of loose‐cell attached voltage clamp recordings on a hM3D(Gq)‐mCherry transduced Purkinje cells, in control condition (up) followed by CNO treatment (0.5 μM, down). (C) Quantification of the mean firing frequency of hM3D(Gq)‐mCherry transduced Purkinje cells in control condition (CTRL) and following addition of CNO. (D, E) Immunohistochemistry showing the expression of ChR2‐YFP (in green) restricted to Purkinje cells (Calb positive, in red) at 4 DIV (D) and 10 DIV (E). (F) Quantification of the percentage of Purkinje cells expressing YFP in the folia with high density of YFP signal (used for analysis). (G) Example of a ChR2‐YFP expressing Purkinje cell (in green, filled white arrowhead) recorded in loose cell‐attached voltage clamp. (H) Representative examples of loose‐cell attached voltage clamp recordings on a ChR2‐YFP expressing Purkinje cells in control slices, without optogenetic stimulation (LED off, up) followed by stimulation at 10 Hz with 10 ms long pulses at 1,5 mW/mm^2^ (down). The pattern of neuronal firing (in black) follows the pattern of light (pulses are indicated with the blue rectangles). (I) Quantification of the mean firing frequency of Purkinje cells without optogenetic stimulation (CTRL) and following optogenetic stimulation (ACT) in myelinated slices. (C, I) Wilcoxon matched‐pairs signed rank test. Each individual point represents the mean for one cell recorded. *n* = 8 cells from 4 animals (C) and *n* = 6 cells from 4 animals (I). (F) *n* = 3 animals per condition. Scale bars: (D, E) 30 μm.

**Figure S6:** Neuronal activity enhancement accelerates myelination of Purkinje cell axons. (A) For the DREADDs approach, cerebellar slices were transduced with AAV8‐hSyn‐hM3D(Gq)‐mCherry after being generated, treated with CNO or DMSO (Ctrl) at 3 DIV for 6 h and fixed 17 h after the end of the stimulation. For the optogenetics approach, L7‐ChR2‐YFP mouse cerebellar slices were stimulated (470 nm, STIM) or not (Ctrl) for 6 h at 3 DIV and fixed 17 h after the end of the stimulation. (B) Purkinje cells (Calbindin, cyan) of slices transduced with DREADDs‐expressing AAV (mCherry, red) are more myelinated (PLP, gray) in CNO‐treated slices compared to control condition. (C) Percentage of Purkinje cell axonal area covered with myelin in activated (CNO, 0.5 μM) versus control condition (Ctrl). (D) Purkinje cells (Calbindin, Cyan) of L7‐ChR2‐YFP cultured cerebellar slices (YFP, green) are more myelinated (PLP, gray) following stimulation (STIM) than in the control condition. (E) Percentage of Purkinje cell axonal area covered with myelin following stimulation (470 nm, STIM) compared to control condition (CTRL). (C) *n* = 6 animals, Paired t test. (E) *n* = 6 animals, Paired t‐test. (B, D) Scale Bar: 20 μm.

**Figure S7:** The inhibition of glutamatergic transmission decreases Nav1.1 expression and node‐like cluster formation ex vivo during myelination. (A) Representative immunostainings of cerebellar slices showing Purkinje cells labeled for Calbindin (blue), AnkyrinG (AnkG, red) and Nav1.1 (green). Following treatment with kynurenic acid (KYN, 1 mM), Purkinje cells exhibit a reduced or absent Nav1.1 expression (green) at the axon initial segment (AIS, visualized by AnkyrinG staining, red) compared to control slices at the onset of myelination. White arrowheads indicate AISs with detectable Nav1.1 signal. (B) Quantification of the percentage of Purkinje cells displaying Nav1.1 at their AIS following KYN treatment. Slices were fixed at 3 days in vitro (DIV), corresponding to the onset of myelination. Each data point represents one animal (*n* = 5 per condition). Paired t‐test. Scale bars: (A) 10 μm (C) Immunostaining of cerebellar slices showing Purkinje cells (Calbindin, blue) with node like‐clusters (Nav, red, not associated to myelin, PLP, white). Following kynurenic acid treatment (KYN, 1 mM), fewer Purkinje cells assemble node‐like clusters compared to control condition. Node‐like clusters are indicated by white arrowheads. (D) Quantification of the percentage of Purkinje cells with node‐like clusters following kynurenic acid treatment (KYN). The slices were fixed at 3 DIV at the onset of myelination. Each point corresponds to one animal. *n* = 6 animals per condition. Paired t test. (C) Scale bar: 20 μm.

**Figure S8:** Validation of the in vivo DREADDs approach coupled to focal demyelination of mouse spinal cord. (A) Schematic representation of the retrograde virus injection in the mouse dorsal spinal cord, followed 4 weeks later by focal demyelination induction. (B) Illustration of the motor cortex from a mouse transduced with AAVrg‐hSyn‐hM3Gq‐mCherry showing cells expressing the hM3Gq receptor (mCherry, red). (C) Mouse corticospinal neurons transduced with AAVrg‐hSyn‐hM3D(Gq)‐mCherry (left panel) or AAVrg‐hSyn‐hM4D(Gi)‐mCherry (right panel) showing an increase or a decrease of cFos expression (in green) in neurons expressing hM3D(Gq) or hM4D(Gi) respectively (mCherry, red) following CNO injection compared to control condition (the spinal cord tissue was collected 1 h after CNO or NaCl injection). mCherry+ cells expressing cFos are indicated by arrowheads. (D‐E) Quantification of the percentage of mCherry+ neurons expressing cFos in mouse transduced with AAVrg‐hSyn‐hM3D(Gq)‐mCherry (D) or AAVrg‐hSyn‐hM4D(Gi)‐mCherry (E). Each point corresponds to one animal. (D) *n* = 4 animals, Mann–Whitney test. (E) *n* = 4 animals for NaCl condition and *n* = 6 for CNO condition, Mann–Whitney test. Scale bars: (B) 1 mm; (C) left panels: 30 μm, right panels: 20 μm.

**Table S1:** Statistical table.

## Data Availability

The data that support the findings of this study are available from the corresponding author upon reasonable request.
