# Supplementary figures and images for: Neuronal Activity Promotes Node‐Like Cluster Assembly Prior to Myelination and Remyelination in the Central Nervous System
Source: Glia. 2026 Feb 23;74(4):e70138. doi: 10.1002/glia.70138 (PMC12929708; doi:10.1002/glia.70138)

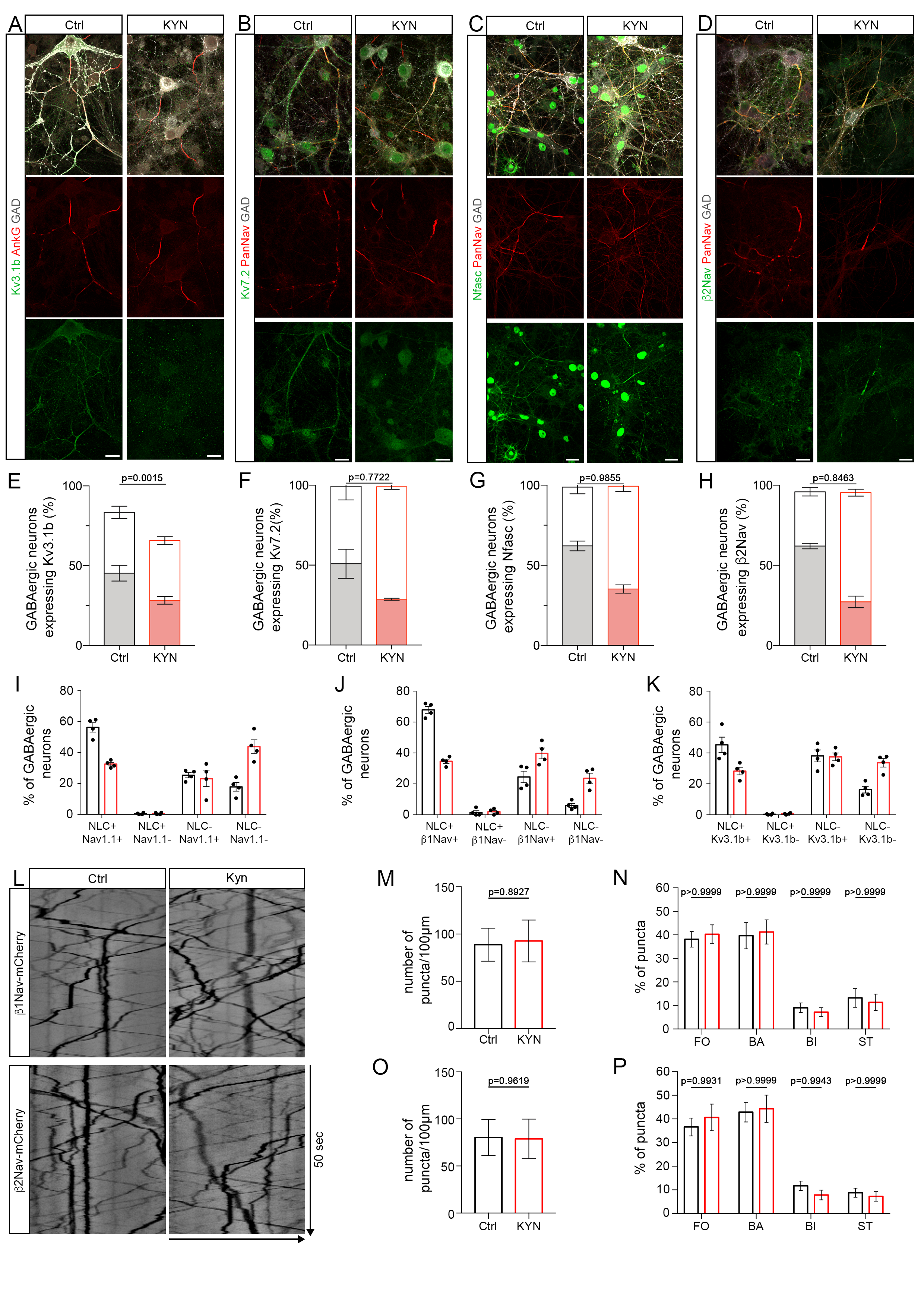

Supplement: Supplementary file 1 — Figure S1: The inhibition of glutamatergic receptors affects the expression of some nodal protein, but does not impact nodal marker axonal transport. The inhibition of glutamatergic inputs in mixed hippocampal cultures by the addition of a glutamatergic antagonist (kynurenic acid, KYN, 1 mM) affects the expression in GABAergic neurons (GAD+, white) of Kv3.1b (A, E), while Kv7.2 (B, F), Nfasc (C, G) and β2Nav (D, H) are still expressed in the vast majority of GABAergic neurons. Of note, at 17 DIV, in control condition, Kv7.2 was restricted to the axon initial segment and node‐like clusters when present. Kv3.1b, though strongly expressed in some GAD+ neurons, was not restricted to, nor enriched at axonal domains. (E‐H) Percentages of GAD+ neurons expressing the nodal marker of interest (percentage of cells with node‐like clusters in dark and of cells without clusters in white) in control condition or following treatment by KYN. Unpaired t‐test. Scale bars: 20 μm. (I‐K) Expression (+ or ‐) of Nav1.1 (I), β1Nav (J) and Kv3.1b (K), in GABAergic neurons with or without clusters (NLC + or—respectively) in control condition (black bars) or following kynurenic acid treatment (red bars). (L) Representative kymographs illustrating β1Nav‐ and β2Nav‐mCherry axonal transport at 17 DIV following kynurenic acid treatment (KYN) compared to control condition. Anterograde transport from left to right. (M‐P) Quantification of the total number of mCherry+ puncta per 100 μm (M, O) and mCherry+ puncta category distribution (N, P) for β1Nav‐ and β2Nav‐mCherry following KYN (red bars) treatment compared to control (black bars). FO: forward, BA: backward and BI: bidirectional moving puncta. ST: stationary puncta. The histograms show the means ± SEM. (M‐O) Unpaired t test, (N, P) Two‐way ANOVA followed by Tukey's multiple comparisons test. β1Nav‐mCherry: n = 4 experiments; β2Nav‐mCherry: n = 3 experiments. (L) Scale bar: 5 s. [file GLIA-74-0-s004.tif]

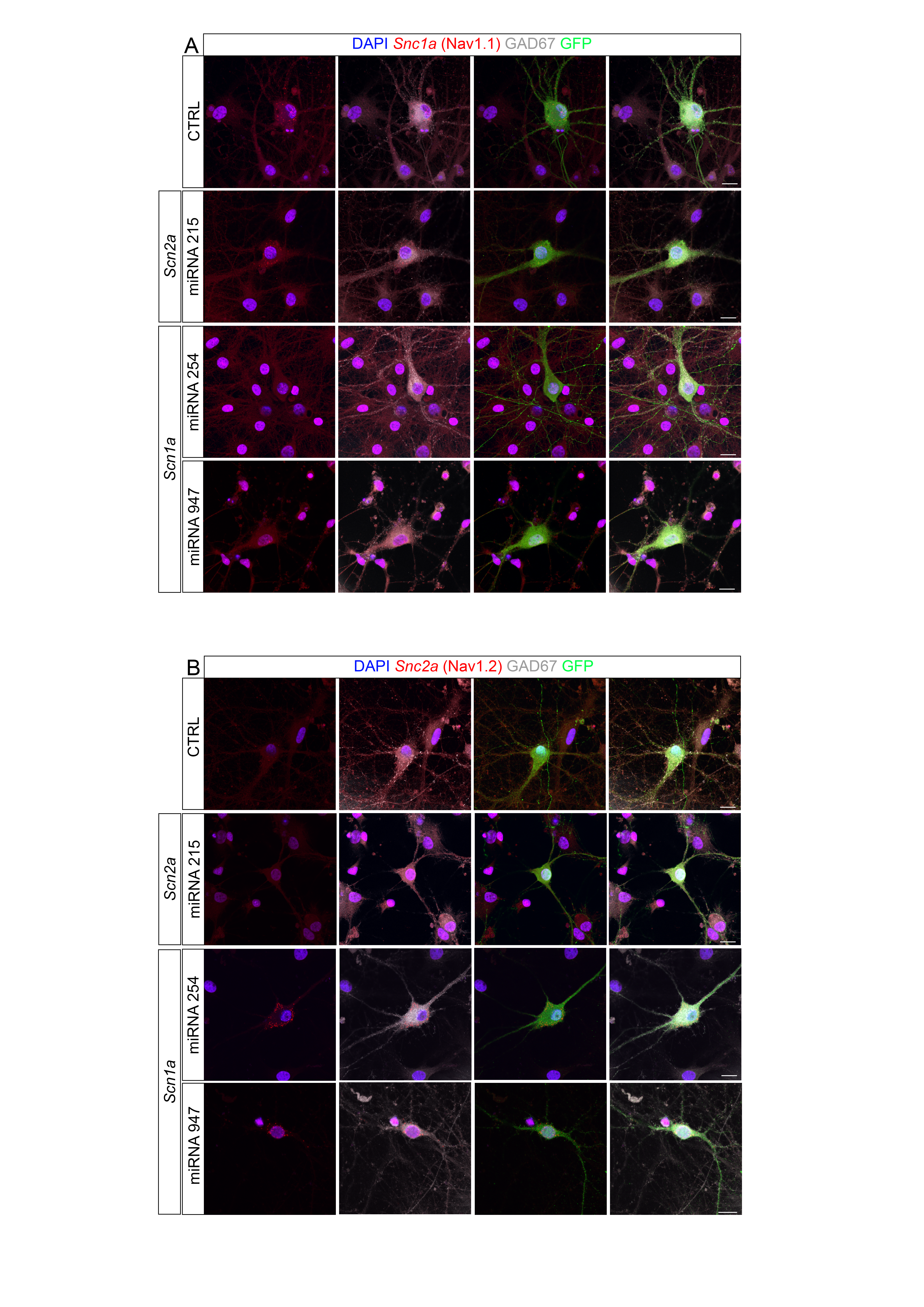

Supplement: Supplementary file 3 — Figure S2: Selective knockdown of Scn1a and Scn2a expression using miRNA‐based silencing in transfected GABAergic neurons. (A) RNAscope combined with immunostaining in mixed hippocampal cultures reveals a strong reduction of Nav1.1 encoding mRNA expression (Scn1a, red), in transfected GABAergic neurons (GAD+, white; emGFP+, green) expressing miR 254 or miR 947, both targeting Scn1a mRNA, compared to those expressing miR Control or miR 215 (targeting Scn2a RNA), with a strong decrease in red puncta observed in the cell body of GAD+/emGFP+ neurons. (B) RNAscope detection of Nav1.2 encoding mRNA (Scn2a, red) shows a strong reduction of Scn2a mRNA in transfected GAD+/emGFP+ GABAergic neurons expressing miR 215 compared to miR Control, confirming its efficacy in silencing Scn2a expression. We further observed that GABAergic neurons expressing miR 254 or miR 947 (targeting Scn1a) exhibit a strong increase in Scn2a mRNA signal, as observed with a strong reinforcement of red puncta in their cell body, suggesting a compensatory upregulation of Scn2A expression in response to Scn1a kock‐down. Scale bars: 10 μm. [file GLIA-74-0-s002.tif]

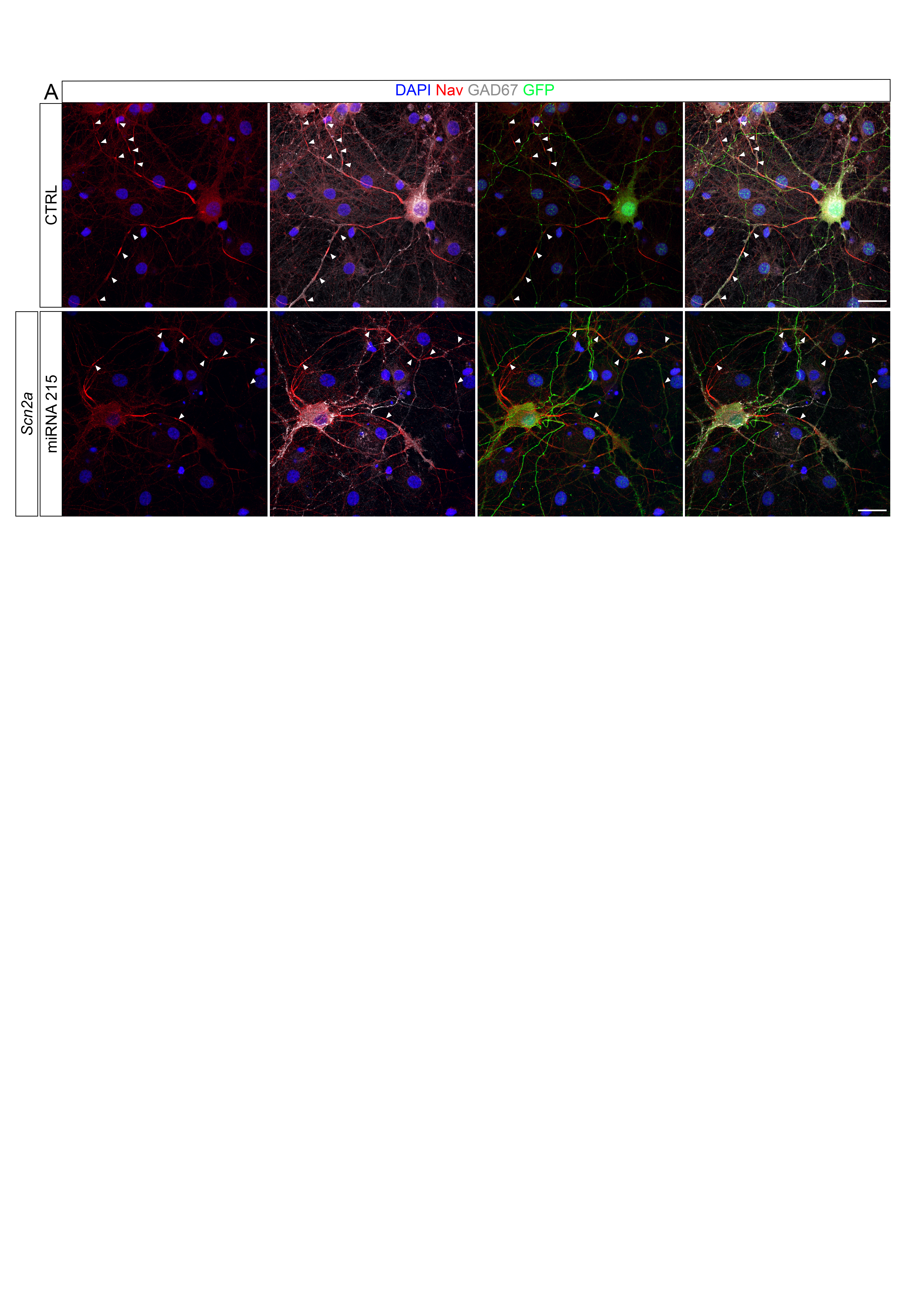

Supplement: Supplementary file 4 — Figure S3: glia70138‐sup‐0004‐FigureS3.tif. Scn2a downregulation does not alter node‐like cluster formation. At DIV17, node‐like cluster assembly (Nav, in red; white arrowheads) is observed in GABAergic neurons (GAD, in white) expressing control miRNA (green, upper lane) and Scn2a‐targetting miRNA 215 (green, lower lane). Scale bars: 30 μm. [file GLIA-74-0-s009.tif]

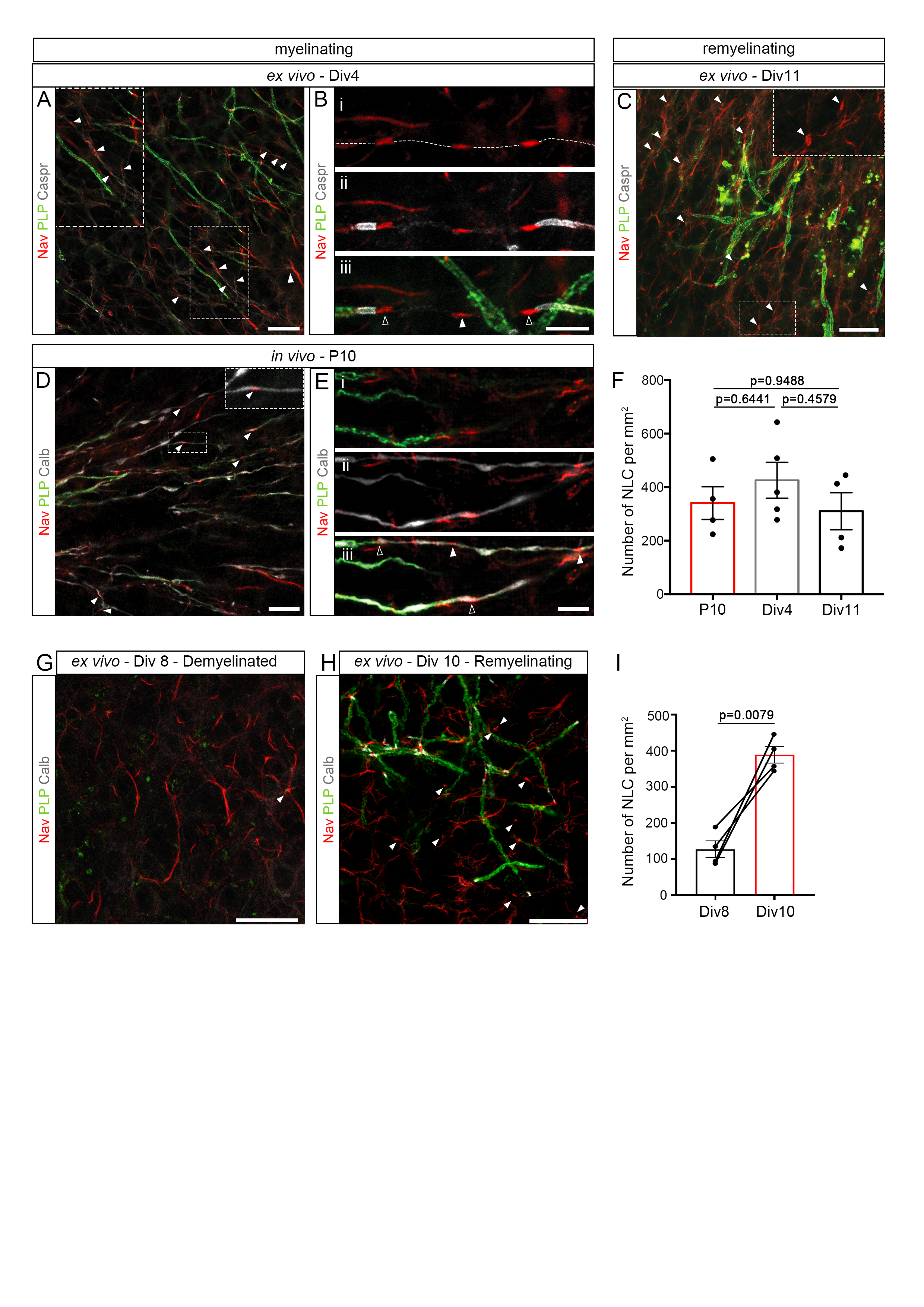

Supplement: Supplementary file 5 — Figure S4: Node‐like clusters are formed prior to myelination and remyelination along Purkinje cells axons. (A) Immunohistostainings of a cerebellar cultured slice at 4 DIV showing node‐like clusters (Nav in red, filled arrowheads) without paranodal clustering (gray, Caspr), distributed in regions with ongoing myelination (PLP, in green). The diffused Caspr signal allows to follow unmyelinated Purkinje axons. (B) Example of an isolated node‐like cluster (Nav in red, filled arrowhead) surrounded by two heminodes (contour arrowheads) along the same axons. (C) Orthogonal projections showing isolated node‐like clusters (Nav in red, filled arrowhead) without paranodal clustering (gray, Caspr) in a remyelinating (PLP, in green) cerebellar slice at 11 DIV. (D) Orthogonal projection of a sagittal section of the cerebellum at P10 showing Purkinje cells axons (Calbindin, in gray), with node‐like clusters (Nav in red, filled arrowhead) along unmyelinated part of their axons (myelin stained by PLP, green). (E) Orthogonal projection showing at a higher magnification two node‐like clusters (Nav in red, filled arrowhead) along an unmyelinated portion of the same axon and heminodes (contour arrowhead) at the extremity of the myelin sheathes. (F) Quantifications of node‐like clusters in the cerebellum in vivo at P10, ex vivo at 3–4 DIV and ex vivo in remyelinating area at 11 DIV show similar densities of node‐like structures in the regions with ongoing myelin deposition. Each value individually plotted corresponds to 1 animal, in vivo P10 and ex vivo 11 DIV: n = 4 animals, ex vivo 4 DIV: n = 5 animals. One‐way ANOVA. (G‐H) Cultured cerebellar slices at 8 DIV (peak of demyelination, G) and 10 DIV (onset of remyelination, H), showing nodal structures clusters (Nav, red), paranodal regions (Caspr, gray), and myelin (PLP, green). Node‐like clusters are indicated by filled arrowheads. (I) Quantification of node‐like clusters in demyelinated and remyelinating conditions. n = 4 animals in [file GLIA-74-0-s008.tif]

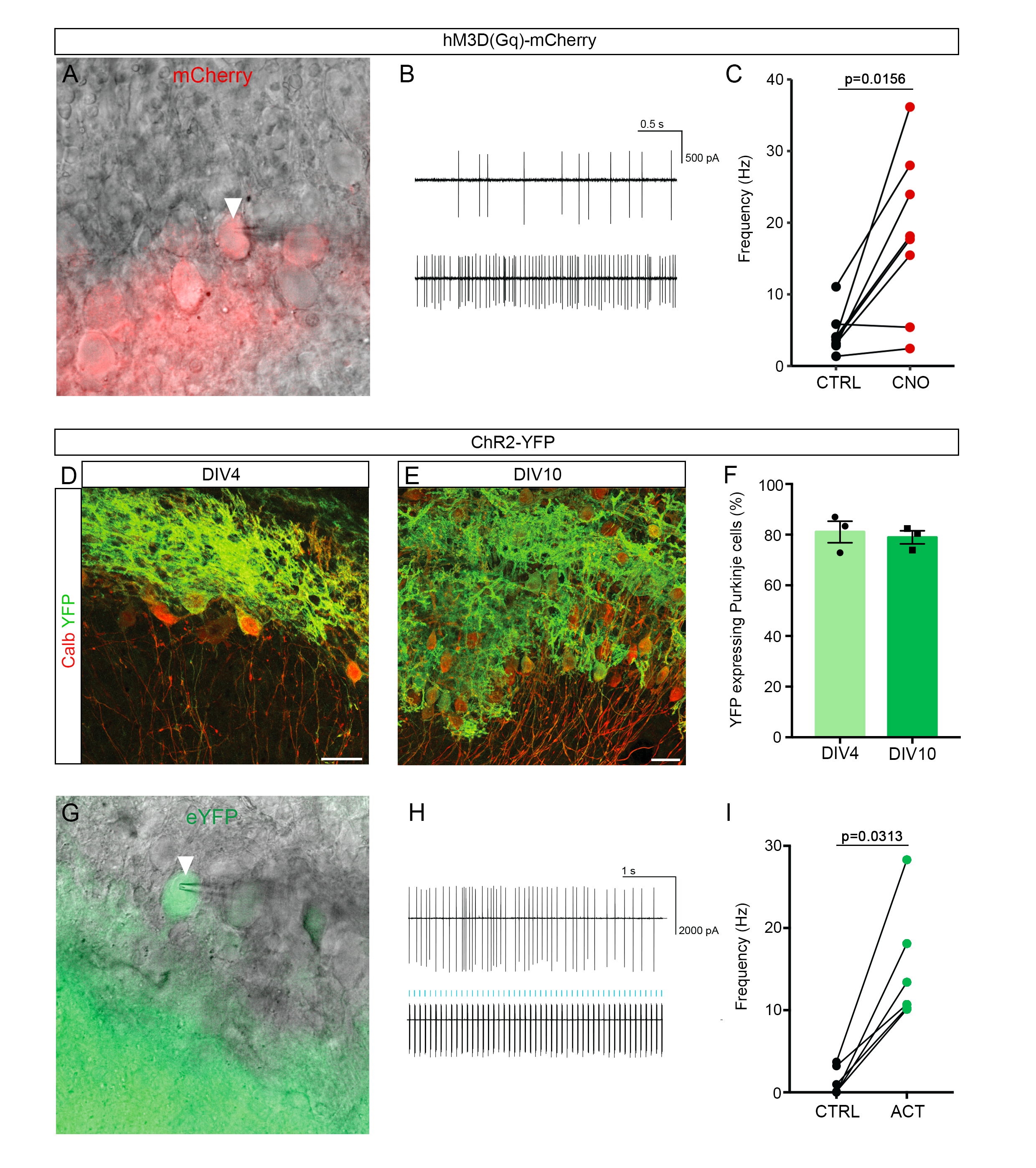

Supplement: Supplementary file 6 — Figure S5: Modulations of the firing activity of Purkinje cells in organotypic cultures of cerebellar slices by DREADD and optogenetic stimulations. (A) Example of a hM3D(Gq)‐mCherry transduced Purkinje cell (filled white arrowhead) recorded in loose cell‐attached voltage clamp. (B) Representative example of loose‐cell attached voltage clamp recordings on a hM3D(Gq)‐mCherry transduced Purkinje cells, in control condition (up) followed by CNO treatment (0.5 μM, down). (C) Quantification of the mean firing frequency of hM3D(Gq)‐mCherry transduced Purkinje cells in control condition (CTRL) and following addition of CNO. (D, E) Immunohistochemistry showing the expression of ChR2‐YFP (in green) restricted to Purkinje cells (Calb positive, in red) at 4 DIV (D) and 10 DIV (E). (F) Quantification of the percentage of Purkinje cells expressing YFP in the folia with high density of YFP signal (used for analysis). (G) Example of a ChR2‐YFP expressing Purkinje cell (in green, filled white arrowhead) recorded in loose cell‐attached voltage clamp. (H) Representative examples of loose‐cell attached voltage clamp recordings on a ChR2‐YFP expressing Purkinje cells in control slices, without optogenetic stimulation (LED off, up) followed by stimulation at 10 Hz with 10 ms long pulses at 1,5 mW/mm2 (down). The pattern of neuronal firing (in black) follows the pattern of light (pulses are indicated with the blue rectangles). (I) Quantification of the mean firing frequency of Purkinje cells without optogenetic stimulation (CTRL) and following optogenetic stimulation (ACT) in myelinated slices. (C, I) Wilcoxon matched‐pairs signed rank test. Each individual point represents the mean for one cell recorded. n = 8 cells from 4 animals (C) and n = 6 cells from 4 animals (I). (F) n = 3 animals per condition. Scale bars: (D, E) 30 μm. [file GLIA-74-0-s010.tif]

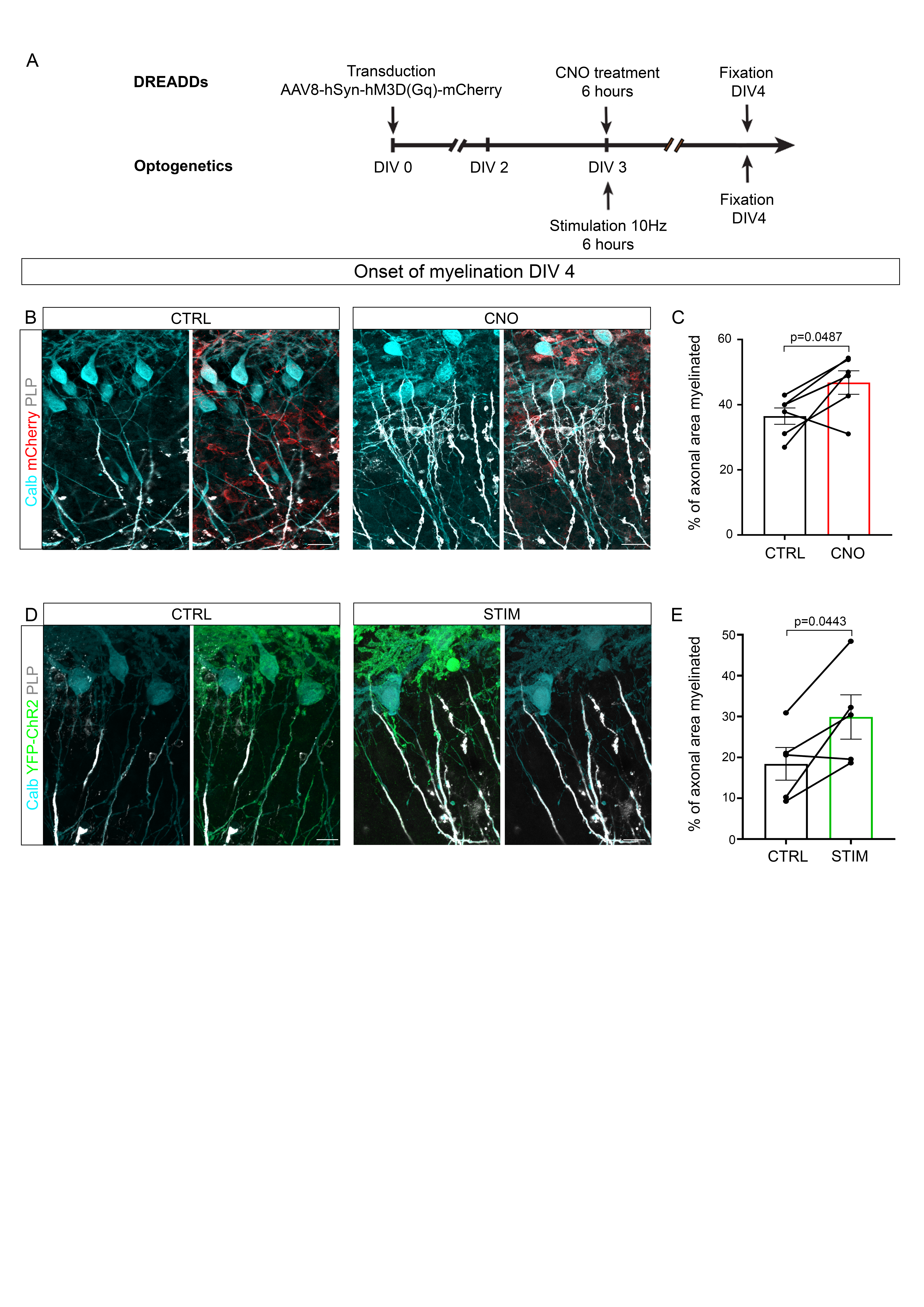

Supplement: Supplementary file 7 — Figure S6: Neuronal activity enhancement accelerates myelination of Purkinje cell axons. (A) For the DREADDs approach, cerebellar slices were transduced with AAV8‐hSyn‐hM3D(Gq)‐mCherry after being generated, treated with CNO or DMSO (Ctrl) at 3 DIV for 6 h and fixed 17 h after the end of the stimulation. For the optogenetics approach, L7‐ChR2‐YFP mouse cerebellar slices were stimulated (470 nm, STIM) or not (Ctrl) for 6 h at 3 DIV and fixed 17 h after the end of the stimulation. (B) Purkinje cells (Calbindin, cyan) of slices transduced with DREADDs‐expressing AAV (mCherry, red) are more myelinated (PLP, gray) in CNO‐treated slices compared to control condition. (C) Percentage of Purkinje cell axonal area covered with myelin in activated (CNO, 0.5 μM) versus control condition (Ctrl). (D) Purkinje cells (Calbindin, Cyan) of L7‐ChR2‐YFP cultured cerebellar slices (YFP, green) are more myelinated (PLP, gray) following stimulation (STIM) than in the control condition. (E) Percentage of Purkinje cell axonal area covered with myelin following stimulation (470 nm, STIM) compared to control condition (CTRL). (C) n = 6 animals, Paired t test. (E) n = 6 animals, Paired t‐test. (B, D) Scale Bar: 20 μm. [file GLIA-74-0-s003.tif]

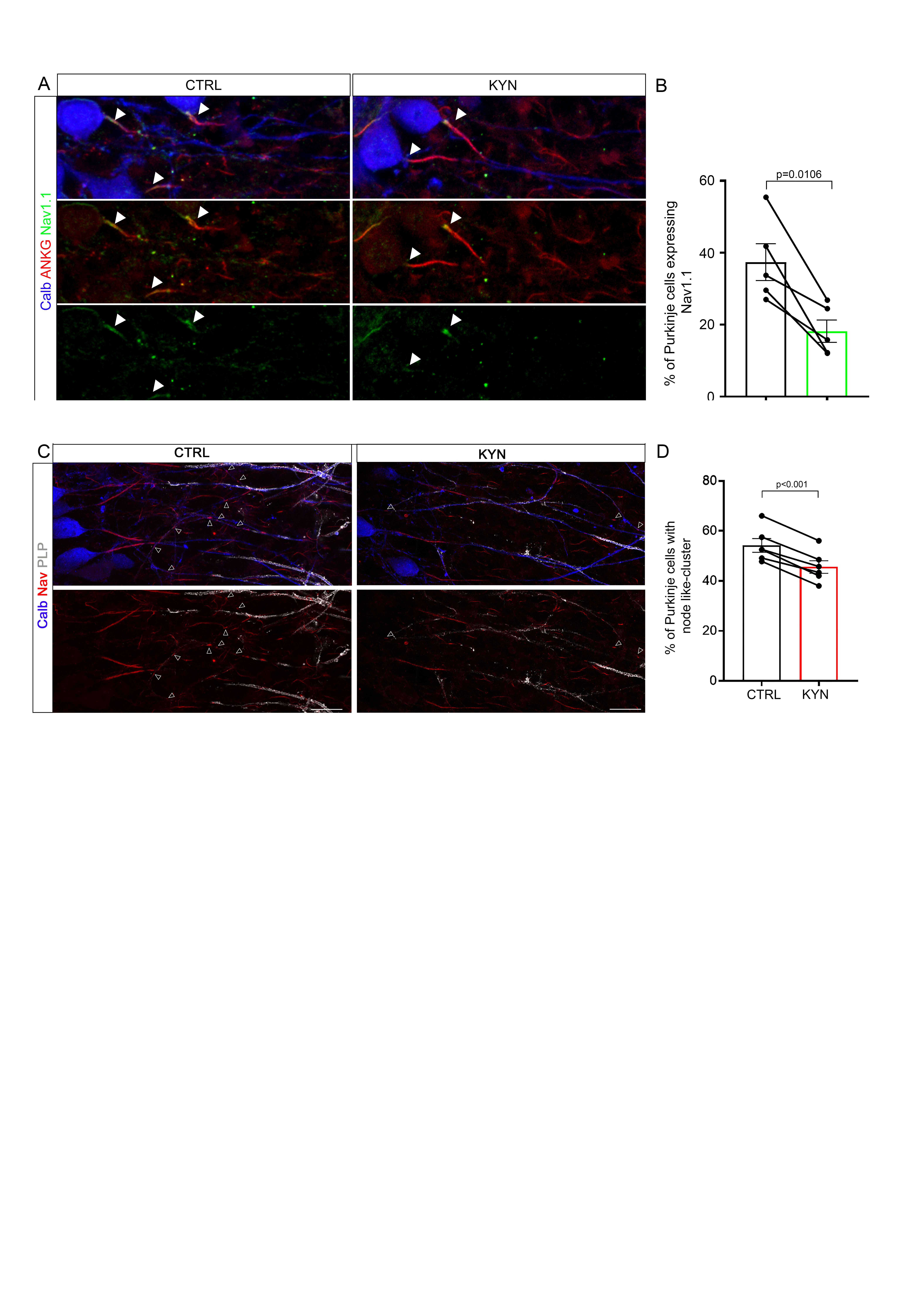

Supplement: Supplementary file 8 — Figure S7: The inhibition of glutamatergic transmission decreases Nav1.1 expression and node‐like cluster formation ex vivo during myelination. (A) Representative immunostainings of cerebellar slices showing Purkinje cells labeled for Calbindin (blue), AnkyrinG (AnkG, red) and Nav1.1 (green). Following treatment with kynurenic acid (KYN, 1 mM), Purkinje cells exhibit a reduced or absent Nav1.1 expression (green) at the axon initial segment (AIS, visualized by AnkyrinG staining, red) compared to control slices at the onset of myelination. White arrowheads indicate AISs with detectable Nav1.1 signal. (B) Quantification of the percentage of Purkinje cells displaying Nav1.1 at their AIS following KYN treatment. Slices were fixed at 3 days in vitro (DIV), corresponding to the onset of myelination. Each data point represents one animal (n = 5 per condition). Paired t‐test. Scale bars: (A) 10 μm (C) Immunostaining of cerebellar slices showing Purkinje cells (Calbindin, blue) with node like‐clusters (Nav, red, not associated to myelin, PLP, white). Following kynurenic acid treatment (KYN, 1 mM), fewer Purkinje cells assemble node‐like clusters compared to control condition. Node‐like clusters are indicated by white arrowheads. (D) Quantification of the percentage of Purkinje cells with node‐like clusters following kynurenic acid treatment (KYN). The slices were fixed at 3 DIV at the onset of myelination. Each point corresponds to one animal. n = 6 animals per condition. Paired t test. (C) Scale bar: 20 μm. [file GLIA-74-0-s005.tif]

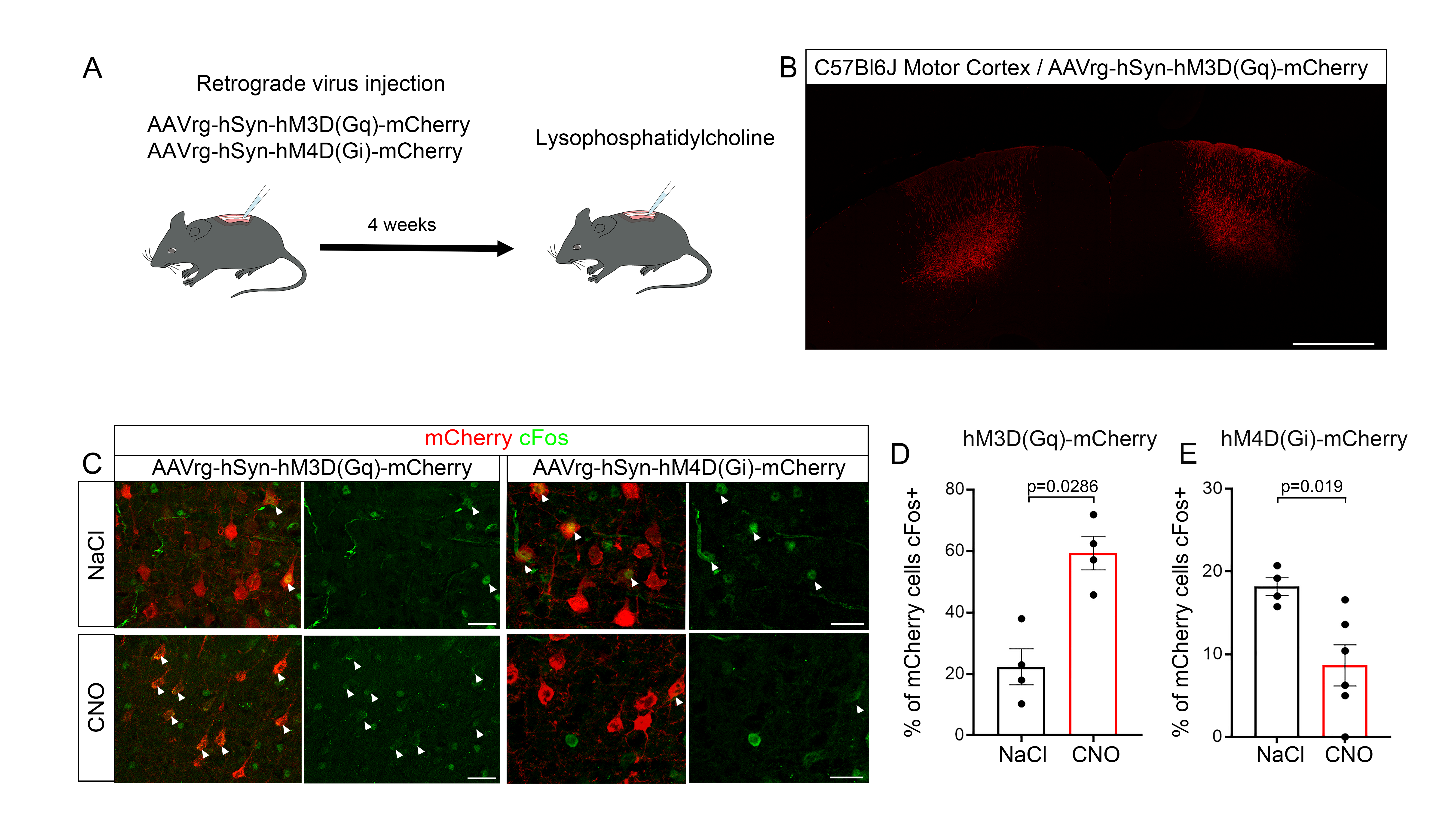

Supplement: Supplementary file 9 — Figure S8: Validation of the in vivo DREADDs approach coupled to focal demyelination of mouse spinal cord. (A) Schematic representation of the retrograde virus injection in the mouse dorsal spinal cord, followed 4 weeks later by focal demyelination induction. (B) Illustration of the motor cortex from a mouse transduced with AAVrg‐hSyn‐hM3Gq‐mCherry showing cells expressing the hM3Gq receptor (mCherry, red). (C) Mouse corticospinal neurons transduced with AAVrg‐hSyn‐hM3D(Gq)‐mCherry (left panel) or AAVrg‐hSyn‐hM4D(Gi)‐mCherry (right panel) showing an increase or a decrease of cFos expression (in green) in neurons expressing hM3D(Gq) or hM4D(Gi) respectively (mCherry, red) following CNO injection compared to control condition (the spinal cord tissue was collected 1 h after CNO or NaCl injection). mCherry+ cells expressing cFos are indicated by arrowheads. (D‐E) Quantification of the percentage of mCherry+ neurons expressing cFos in mouse transduced with AAVrg‐hSyn‐hM3D(Gq)‐mCherry (D) or AAVrg‐hSyn‐hM4D(Gi)‐mCherry (E). Each point corresponds to one animal. (D) n = 4 animals, Mann–Whitney test. (E) n = 4 animals for NaCl condition and n = 6 for CNO condition, Mann–Whitney test. Scale bars: (B) 1 mm; (C) left panels: 30 μm, right panels: 20 μm. [file GLIA-74-0-s006.tif]
