# Supplementary material for: Neuronal Activity Promotes Node‐Like Cluster Assembly Prior to Myelination and Remyelination in the Central Nervous System
Source: Glia. 2026 Feb 23;74(4):e70138. doi: 10.1002/glia.70138 (PMC12929708; doi:10.1002/glia.70138)
Supplement: Supplementary file 2 — Appendix S1: Supporting Information. [file GLIA-74-0-s001.docx]

**SUPPLEMENTARY INFORMATIONS**

**Figure S1. The inhibition of glutamatergic receptors affects the expression of some nodal protein, but does not impact nodal marker axonal transport.** The inhibition of glutamatergic inputs in mixed hippocampal cultures by the addition of a glutamatergic antagonist (kynurenic acid, KYN, 1mM) affects the expression in GABAergic neurons (GAD+, white) of Kv3.1b (A, E), while Kv7.2 (B, F), Nfasc (C, G) and β2Nav (D, H) are still expressed in the vast majority of GABAergic neurons. Of note, at 17 DIV, in control condition, Kv7.2 was restricted to the axon initial segment and node-like clusters when present. Kv3.1b, though strongly expressed in some GAD+ neurons, was not restricted to, nor enriched at axonal domains. (E-H) Percentages of GAD+ neurons expressing the nodal marker of interest (percentage of cells with node-like clusters in dark and of cells without clusters in white) in control condition or following treatment by KYN. Unpaired t-test. Scale bars: 20 μm. (I-K) Expression (+ or -) of Nav1.1 (I), β1Nav (J) and Kv3.1b (K), in GABAergic neurons with or without clusters (NLC + or – respectively) in control condition (black bars) or following kynurenic acid treatment (red bars). (L) Representative kymographs illustrating β1Nav- and β2Nav-mCherry axonal transport at 17 DIV following kynurenic acid treatment (KYN) compared to control condition. Anterograde transport from left to right. (M-P) Quantification of the total number of mCherry+ puncta per 100 μm (M, O) and mCherry+ puncta category distribution (N, P) for β1Nav- and β2Nav-mCherry following KYN (red bars) treatment compared to control (black bars). FO: forward, BA: backward and BI: bidirectional moving puncta. ST: stationary puncta. The histograms show the means ± SEM. (M-O) Unpaired t test, (N, P) Two-way ANOVA followed by Tukey's multiple comparisons test. β1Nav-mCherry: n=4 experiments; β2Nav-mCherry: n=3 experiments. (L) Scale bar: 5s.

**Figure S2. Selective knockdown of Scn1a and Scn2a expression using miRNA-based silencing in transfected GABAergic neurons.** (A) RNAscope combined with immunostaining in mixed hippocampal cultures reveals a strong reduction of Nav1.1 encoding mRNA expression (Scn1a, red), in transfected GABAergic neurons (GAD+, white; emGFP+, green) expressing miR 254 or miR 947, both targeting Scn1a mRNA, compared to those expressing miR Control or miR 215 (targeting Scn2a RNA), with a strong decrease in red puncta observed in the cell body of GAD+/emGFP+ neurons. (B) RNAscope detection of Nav1.2 encoding mRNA (Scn2a, red) shows a strong reduction of Scn2a mRNA in transfected GAD+/emGFP+ GABAergic neurons expressing miR 215 compared to miR Control, confirming its efficacy in silencing Scn2a expression. We further observed that GABAergic neurons expressing miR 254 or miR 947 (targeting Scn1a) exhibit a strong increase in Scn2a mRNA signal, as observed with a strong reinforcement of red puncta in their cell body, suggesting a compensatory upregulation of *Scn2A* expression in response to *Scn1a* kock-down. Scale bars: 10 μm.

**Figure S3. *Scn2a* downregulation does not alter node-like cluster formation.**

At DIV17, node-like cluster assembly (Nav, in red; white arrowheads) is observed in GABAergic neurons (GAD, in white) expressing control miRNA (green, upper lane) and *Scn2a-*targetting miRNA 215 (green, lower lane). Scale bars: 30 μm.

**Figure S4. Node-like clusters are formed prior to myelination and remyelination along Purkinje cells axons.** (A) Immunohistostainings of a cerebellar cultured slice at 4 DIV showing node-like clusters (Nav in red, filled arrowheads) without paranodal clustering (gray, Caspr), distributed in regions with ongoing myelination (PLP, in green). The diffused Caspr signal allows to follow unmyelinated Purkinje axons. (B) Example of an isolated node-like cluster (Nav in red, filled arrowhead) surrounded by two heminodes (contour arrowheads) along the same axons. (C) Orthogonal projections showing isolated node-like clusters (Nav in red, filled arrowhead) without paranodal clustering (gray, Caspr) in a remyelinating (PLP, in green) cerebellar slice at 11 DIV. (D) Orthogonal projection of a sagittal section of the cerebellum at P10 showing Purkinje cells axons (Calbindin, in gray), with node-like clusters (Nav in red, filled arrowhead) along unmyelinated part of their axons (myelin stained by PLP, green). (E) Orthogonal projection showing at a higher magnification two node-like clusters (Nav in red, filled arrowhead) along an unmyelinated portion of the same axon and heminodes (contour arrowhead) at the extremity of the myelin sheathes. (F) Quantifications of node-like clusters in the cerebellum *in vivo* at P10, *ex vivo* at 3-4 DIV and *ex vivo* in remyelinating area at 11 DIV show similar densities of node-like structures in the regions with ongoing myelin deposition. Each value individually plotted corresponds to 1 animal, *in vivo* P10 and *ex vivo* 11 DIV: n=4 animals, *ex vivo* 4 DIV: n=5 animals. One-way ANOVA. **(G-H)** Cultured cerebellar slices at 8 DIV (peak of demyelination, G) and 10 DIV (onset of remyelination, H), showing nodal structures clusters (Nav, red ), paranodal regions (Caspr, gray), and myelin (PLP, green). Node-like clusters are indicated by filled arrowheads. (I) Quantification of node-like clusters in demyelinated and remyelinating conditions. n=4 animals in each condition. Paired t-test. Scale bars: (A, C) 20 μm, (B,D) 5 μm, (D) 10 μm, (G,H) 15 µm.

**Figure S5. Modulations of the firing activity of Purkinje cells in organotypic cultures of cerebellar slices by DREADD and optogenetic stimulations.** (A) Example of a hM3D(Gq)-mCherry transduced Purkinje cell (filled white arrowhead) recorded in loose cell-attached voltage clamp. (B) Representative example of loose-cell attached voltage clamp recordings on a hM3D(Gq)-mCherry transduced Purkinje cells, in control condition (up) followed by CNO treatment (0.5 μM, down). (C) Quantification of the mean firing frequency of hM3D(Gq)-mCherry transduced Purkinje cells in control condition (CTRL) and following addition of CNO. (D, E) Immunohistochemistry showing the expression of ChR2-YFP (in green) restricted to Purkinje cells (Calb positive, in red) at 4 DIV (D) and 10 DIV (E). (F) Quantification of the percentage of Purkinje cells expressing YFP in the folia with high density of YFP signal (used for analysis). (G) Example of a ChR2-YFP expressing Purkinje cell (in green, filled white arrowhead) recorded in loose cell-attached voltage clamp. (H) Representative examples of loose-cell attached voltage clamp recordings on a ChR2-YFP expressing Purkinje cells in control slices, without optogenetic stimulation (LED off, up) followed by stimulation at 10Hz with 10ms long pulses at 1,5mW/mm^2^ (down). The pattern of neuronal firing (in black) follows the pattern of light (pulses are indicated with the blue rectangles). (I) Quantification of the mean firing frequency of Purkinje cells without optogenetic stimulation (CTRL) and following optogenetic stimulation (ACT) in myelinated slices. (C, I) Wilcoxon matched-pairs signed rank test. Each individual point represents the mean for one cell recorded. n=8 cells from 4 animals (C) and n=6 cells from 4 animals (I). (F) n=3 animals per condition. Scale bars: (D, E) 30 μm.

**Figure S6. Neuronal activity enhancement accelerates myelination of Purkinje cell axons.**

(A) For the DREADDs approach, cerebellar slices were transduced with AAV8-hSyn-hM3D(Gq)-mCherry after being generated, treated with CNO or DMSO (Ctrl) at 3 DIV for 6 hours and fixed 17 hours after the end of the stimulation. For the optogenetics approach, L7-ChR2-YFP mouse cerebellar slices were stimulated (470nm, STIM) or not (Ctrl) for 6 hours at 3 DIV and fixed 17 hours after the end of the stimulation. (B) Purkinje cells (Calbindin, cyan) of slices transduced with DREADDs-expressing AAV (mCherry, red) are more myelinated (PLP, grey) in CNO-treated slices compared to control condition. (C) Percentage of Purkinje cell axonal area covered with myelin in activated (CNO, 0.5 μM) versus control condition (Ctrl). (D) Purkinje cells (Calbindin, Cyan) of L7-ChR2-YFP cultured cerebellar slices (YFP, green) are more myelinated (PLP, grey) following stimulation (STIM) than in the control condition. (E) Percentage of Purkinje cell axonal area covered with myelin following stimulation (470nm, STIM) compared to control condition (CTRL). (C) n = 6 animals, Paired t test. (E) n=6 animals, Paired t-test. (B, D) Scale Bar: 20 μm.

**Figure S7. The inhibition of glutamatergic transmission decreases Nav1.1 expression and node-like cluster formation ex vivo during myelination.**

(A) Representative immunostainings of cerebellar slices showing Purkinje cells labeled for Calbindin (blue), AnkyrinG (AnkG, red) and Nav1.1 (green). Following treatment with kynurenic acid (KYN, 1 mM), Purkinje cells exhibit a reduced or absent Nav1.1 expression (green) at the axon initial segment (AIS, visualized by AnkyrinG staining, red) compared to control slices at the onset of myelination. White arrowheads indicate AISs with detectable Nav1.1 signal. (B) Quantification of the percentage of Purkinje cells displaying Nav1.1 at their AIS following KYN treatment. Slices were fixed at 3 days in vitro (DIV), corresponding to the onset of myelination. Each data point represents one animal (n = 5 per condition). Paired t-test. Scale bars: (A) 10 μm (C) Immunostaining of cerebellar slices showing Purkinje cells (Calbindin, blue) with node like-clusters (Nav, red, not associated to myelin, PLP, white). Following kynurenic acid treatment (KYN, 1mM), fewer Purkinje cells assemble node-like clusters compared to control condition. Node-like clusters are indicated by white arrowheads. (D) Quantification of the percentage of Purkinje cells with node-like clusters following kynurenic acid treatment (KYN). The slices were fixed at 3 DIV at the onset of myelination. Each point corresponds to one animal. n=6 animals per condition. Paired t test. (C) Scale bar: 20 μm.

**Figure S8. Validation of the in vivo DREADDs approach coupled to focal demyelination of mouse spinal cord**

(A) Schematic representation of the retrograde virus injection in the mouse dorsal spinal cord, followed 4 weeks later by focal demyelination induction. (B) Illustration of the motor cortex from a mouse transduced with AAVrg-hSyn-hM3Gq-mCherry showing cells expressing the hM3Gq receptor (mCherry, red). (C) Mouse corticospinal neurons transduced with AAVrg-hSyn-hM3D(Gq)-mCherry (left panel) or AAVrg-hSyn-hM4D(Gi)-mCherry (right panel) showing an increase or a decrease of cFos expression (in green) in neurons expressing hM3D(Gq) or hM4D(Gi) respectively (mCherry, red) following CNO injection compared to control condition (the spinal cord tissue was collected 1h after CNO or NaCl injection). mCherry+ cells expressing cFos are indicated by arrowheads. (D-E) Quantification of the percentage of mCherry+ neurons expressing cFos in mouse transduced with AAVrg-hSyn-hM3D(Gq)-mCherry (D) or AAVrg-hSyn-hM4D(Gi)-mCherry (E). Each point corresponds to one animal. (D) n = 4 animals, Mann-Whitney test. (E) n = 4 animals for NaCl condition and n=6 for CNO condition, Mann-Whitney test. Scale bars: (B) 1mm; (C) left panels: 30 μm, right panels: 20μm.

**SUPPLEMENTARY METHODS**

**Preparation of OCM and purified neuronal cultures supplemented with OCM.**

Glial cell cultures were prepared from cerebral cortices of P2 Wistar rats as described previously^72^. After meninges were removed, cortices were incubated for 35 min in papain (30 U/mL; Worthington), supplemented with L-cysteine (0.24 mg/mL, Sigma) and DNase (50 μg/mL, Worthington) in DMEM at 37°. They were then mechanically dissociated and passed through a 70-μm filter. Cells were resuspended in DMEM Glutamax with 10% FCS and 1% penicillin–streptomycin (100 IU/mL each). After 7–14 days in vitro (DIV), oligodendroglial lineage cells were purified from glial cell cultures which initially contain astrocytes and microglial cells. After cultures were shaken overnight at 230 rpm and 37 °C, overlying oligodendroglial and microglial cells could be selectively detached. Microglia were then eliminated by differential adhesion. Collected cells were incubated in dishes for 15 min. Nonadherent cells were retrieved and centrifuged in DMEM for 5 min at 400G. They were resuspended and seeded at a density of 1.5 × 10^5^/cm^2^ on polyethyleneimine-coated (PEI) dishes with BS medium and 0.5% PDGF. The cells were then incubated for two days in BS medium and then placed in NCM. The medium from these cultures was collected 48h later, filtered (0.22 μm), and stored at 4°C to be used as OCM. Purified neuron cultures (PUR) were prepared by adding anti-mitotic agents (FdU and U 5 μM) for 36h to mixed hippocampal cultures prepared as described above, starting 24 h after dissection. OCM (500 μL/well) was then added to the purified neuron cultures at 3 DIV. One-third of the medium was replaced with NCM at 7 DIV, and then twice a week.

**Reverse Transcriptase quantitative PCR (RT-qPCR)**

To quantify expression of *SCN1A* (encoding Nav1.1), S*CN2A* (encoding Nav1.2) and *SCN1B* (encoding β1Nav) genes by RT-qPCR, we designed the primers using the Primer-Blast (NCBI): SCN1A-FW 5’GGAAGCCGCACAGCAGG3’, SCN1A-RV 5’AGCTATCTGAGAGCCTGCCC3’;

SCN2A-FW 5’ACTGCTACATTCAACACGCAC3’, SCN2A-RV 5’TCCTCGCGTAAGAAAGTGCTGA3’;

SCN1B-FW: 5’TCCCGGACGCGGAGTATC3’, SCN1B-RV 5’CCATACACTGCCTCGGTCTC3’

The following housekeeping gene primers (*TBP* and *GAPDH)* were used: GAPDH-FW 5’CACCATCTTCCAGGAGCGAG3’, GAPDH-RV 5’GGTGGTGAAGACGCCAGTAG3’; TBP-FW 5’CCCCGGTGGAAGACAGTTTTA3’, TBP-RV 5’CACCATGAAACAGTGATGTGGG3’

RNA was isolated from pure hippocampal neuron cultures with OCM or without OCM (Purified) treated or not with kynurenic acid (KYN, final concentration 1mM) using the miRNeasy micro kit protocol (Qiagen, 74004). Briefly, cells were lysed with QIAzol lysis reagent and mix with chloroform, then RNA was purified and quantified. Reverse transcription was done with 500ng RNA with the Maxima first strand cDNA kit for RT-PCR (K1642, Thermofisher/Invitrogen). Then, the qPCR was performed with the LightCycler 480 SYBR green I Master (Roche, 4707516001) in a multiwell plate 96 (Roche, 4729692001). The qPCR reaction was done in triplicate by mixing LC480 SYBR green, the reverse and forward primers 20µM, final concentration 0.3µM, and the cDNA. The real-time PCR program was a preincubation step (95°C, 300sec), then a three-step amplification (95°C, 10 sec, 60°C, 10 sec and 72°C, 10 sec for 45 cycles), then a melting step (95°C, 10 sec, 65°C, 60 sec and 97°C, 1 sec). Relative quantification of qPCR data was done with GAPDH and TBP reference genes as the normalizer by Paffl method (Real-time PCR application guide, 2006, Bio-Rad laboratories).

**Plasmid constructs and cell culture transfection**

The plasmids pTRIPSyn-β1NavmCherry and pTRIPSyn-β2NavmCherry used for the axonal transport study have been previously described ^9^.

The oligonucleotides used to generate the miRNA constructs targeting Nav1.1 are as follow: miR 254:

Top:TGCTGTAACAGGGCATTCACAACCACGTTTTGGCCACTGACTGACGTGGTTGTATGCCCTGTTA; Bottom:CCTGTAACAGGGCATACAACCACGTCAGTCAGTGGCCAAAACGTGGTTGTGAATGCCCTGTTAC

miR 947:

Top:TGCTGAATGCTTGTCACATAATCGCTGTTTTGGCCACTGACTGACAGCGATTATGACAAGCATT; Bottom:CCTGAATGCTTGTCATAATCGCTGTCAGTCAGTGGCCAAAACAGCGATTATGTGACAAGCATTC

miR 215:

Top: TGCTGACCAGAAGTACGTTCATTATGGTTTTGGCCACTGACTGACCATAATGAGTACTTC;

Bottom: CCTGACCAGAAGTACTCATTATGGTCAGTCAGTGGCCAAAACCATAATGAACGTACTTCT

The miRNA plasmids used for knockdown studies were generated following manufacturer's instructions (K493600; ThermoFisher Scientific). The control miRNA construct was provided in the kit. For all the miRNA constructs used, emGFP is co-expressed with the encoded miRNA, allowing the detection of the transfected cells. Transfection of rat primary neurons was performed as previously described^8^, at 6 DIV with a total of 500ng DNA and 1.0 μl Lipofectamine 2000 reagent per well (11668019; ThermoFisher Scientific) in Opti-MEM reduced serum medium (31985062; ThermoFisher Scientific). 50ng/μl of plasmid were used per well for nodal protein expression and 400 ng/μl for miRNA, supplemented to 500ng DNA with pBlueScript vector.

**Electrophysiology recordings**

Organotypic cerebellar slices at 9 to 11 DIV were transferred to a recording chamber and continuously superfused with oxygenated (95% O2 and 5% CO2) aCSF containing (in mM): 124 NaCl, 3 KCl, 1.25 NaH2PO4, 26 NaHCO3, 1.3 MgSO4, 2.5 CaCl2, and 15 glucose (pH 7.4), all from Sigma Aldrich). Purkinje cells were visualized under differential interference contrast optics using a 63X water immersion lens (N.A. 1). Loose cell-attached voltage clamp recordings of the spontaneous firing activity of Purkinje cells were performed at 30-34°C with a borosilicate glass pipette filled with aCSF. Signals were amplified with a Multiclamp 700B amplifier (Molecular devices), sampled and filtered at 10 kHz with a Digidata 1550B (Molecular Devices). Data were acquired with the pClamp software (Molecular devices). To avoid any alteration of the spontaneous firing frequency of the cell by the patch procedure ^73^, the holding membrane potential was set to the value at which zero current was injected by the amplifier. The resistance of the seal (Rseal) was controlled and calculated every minute from the current response to a voltage step (200 ms; -10 mV). Only recordings with a Rseal in the range of 10 to 100MΩ and stable during the recording procedure were included in the analysis. To test the modulation of neuronal activity by DREADDs, recordings were performed in control and the perfusion was then switched to a bath with 0.5μM of N-clozapine (CNO) while recording the same neuron. To test optogenetics stimulation, a LED with an excitation filter 482/35 was calibrated to stimulate the field of view at 1.5mW/mm^2^ and individual neurons were successively recorded with no stimulation and with pulses of 10 millisecond at 10Hz. The mean firing rate was analyzed over 110 seconds recording time window using a threshold crossing spike detection in Clampfit (Molecular devices) and calculated as the number of action potential divided by the duration of the recording.

**Fixation and immunohistochemistry**

*In vitro* *cultured hippocampal neurons*

Cell cultures were fixed at DIV14 or 17 with 4% paraformaldehyde (PFA, Electron Microscopy Services, ThermoFisher Scientific) for 10 min, or with 1% PFA for 10 min at room temperature (RT) and then incubated with methanol for 10 min at −20°C (for αNav staining). After fixation, cells were washed in 1× PBS, before being incubated with blocking solution (1× PBS with 5% Normal Goat Serum [50-062Z; ThermoFisher Scientific], 0.1% Triton X-100) for 15 min and with primary antibodies solutions for 2 to 3 hours at RT. Coverslips were then washed in 1× PBS and incubated with secondary antibodies solutions for 1 hr at RT. Coverslips were washed in 1× PBS and mounted on glass slides with Fluoromount G (Southern Biotech, with or without DAPI).

*Ex vivo* *cultured cerebellar slices*

Cerebellar slices were fixed as described before ^37^, with 4% PFA (Electron Microscopy, ThermoFisher Scientific) for 5 minutes followed by 1% PFA for 25 minutes at RT and washed in PBS. Subsequently, the slices were incubated in absolute ethanol (Sigma Aldrich) at -20°C for 20 minutes and washed in PBS. The slices were blocked for 1 hour in PBS, 5% Normal Goat Serum (50-062Z; ThermoFisher Scientific), 0.3% Triton X-100 (9036-19-5, Sigma) and incubated with primary antibodies diluted in blocking solution overnight at RT. The slices were then washed in PBS, incubated for 3 hours at RT in the dark with secondary antibodies diluted in blocking solution. The slices were washed in PBS and mounted between a glass slide and a coverslip (VWR) with Fluoromount-G (0100-01, Southern Biotech).

*Mouse central nervous tissues fixation and collection*

Adult and P10 mice were perfused with 2% PFA and the brain and spinal cord collected and post-fixed in PFA 2% for 30 minutes, washed in PBS and incubated in PBS with 30% sucrose (S0389, Sigma Aldrich) for 3 days at 4°C for cryoprotection. The tissues were then included in O.C.T (﻿Tissue-Tek, Sakura). Using a cryostat (Leica CM 1950), the brains were cut sagittaly or coronally and the spinal cord cut longitudinally in 30μm and 20µm thick sections respectively. Sections were collected on Superfrost+ glass slides (48311-703, VWR). For immunohistochemistry, the slides were first placed in absolute ethanol at -20°C for 20 minutes. They were then incubated with a blocking solution containing PBS, 5% Normal Goat Serum and 0.2% Triton X-100 for at least 30 minutes at RT. Following PBS washes, the slides were incubated with the primary antibodies diluted in blocking solution overnight at RT and the next day with the secondary antibodies diluted in blocking solution for 2 hours in the dark at RT. The slides were mounted using Fluoromount with or without Hoechst (Southern Biotech) placed on an adapted coverslip (VWR), and left to dry at RT before being stored at 4°C.

**RNascope coupled to immunocytochemistry on mixed hippocampal cell culture**

RNAscope was performed using the RNAscope Multiplex Fluorescent v2 Kit (ACD, #323110) combined with Protease III (ACD, #322381) and probes targeting Scn1a (ACD, #411281-C2) and Scn2a (ACD, #411291-C3) on mixed hippocampal cell cultures transfected with miRNA-GFP plamsids. Cell cultures were fixed at DIV14 with 4% paraformaldehyde and dehydrated (50%, 70% and 100% ethanol sequentially) before storage at –20 °C. Prior to hybridization, cultures were rehydrated (70% and 50% ethanol sequentially, followed by two washes in PBS). Cell cultures were then incubated in blocking buffer (PBS1x, 5% normal goat serum (ThermoFisher, #50-062Z), 0.1% Triton X-100) for 15 minutes, followed by overnight incubation with anti-GFP and GAD67 primary antibodies at room temperature. After primary antibodies incubation, cultures were treated with 4% PFA for 5 minutes and washed in PBS. Protease treatment was performed by incubating cells with Protease III (1:15 in PBS) for 10 minutes at 40 °C. Probe hybridization was carried out for 1 hour at 40 °C. Amplification steps were performed sequentially at 40 °C: Amp1 for 30 minutes, Amp2 for 20 minutes, and Amp3 for 15 minutes. The cells were next incubated with HRP-C2 or HRP-C3 reagents, for 15 minutes at 40 °C, with a final incubation with Opal 550 (PerkinElmer, FP1496001KT; 1:1000 in RNAscope TSA Buffer, ACD #322809) for 30 minutes at 40 °C, followed by incubation with RNAscope FL v2 Blocker for 15 minutes at 40 °C. Incubation with secondary antibodies (anti-chicken coupled to A488 and anti-mouse IgG2A coupled 4647) and coverslips mounting were then performed as described above.

**Antibodies**

The following primary antibodies were used: mouse IgG2a anti-AnkyrinG (clone N106/36; 1:100, Neuromab), mouse IgG2b anti-AnkyrinG (1:75; clone N106/65, NeuroMAB), rabbit anti-AnkG (1:300, custom anti-peptide polyclonal antibody, targeted against LLERSSITMTPPASPKSN sequence, Eurogentech), mouse IgG1 anti-Pan Nav (1:150-1:300, Sigma), mouse IgG1 anti-Nav1.1 (1:100, clone K74/71, NeuroMAB), rabbit anti-Kv7.2 (1/250, 368.103, Synaptic System), mouse IgG1 anti Kv3.1b (1:100, clone N16b8, NeuroMAB), rabbit anti-β1Nav (1:100; kindly provided by Pr P.J. Brophy, University of Edinburgh, UK), rabbit anti-β2Nav (1:250; Millipore), rabbit anti-Nfasc (pan; 1:100; Abcam), rabbit anti-Caspr (1:300, Abcam), ﻿mouse anti-Calbindin (1:500; Sigma), rabbit anti-Calbindin (1:300; Swant), rat anti-PLP (1:10; hybridoma, kindly provided by Dr. K. Ikenaka, Okasaki, Japan), chicken anti-GFP (1:250, Millipore), chicken anti-mCherry (1:1000 to 1:2000, EnCor Biotechnology), mouse IgG2a anti-GAD67 (clone 1G10.2; 1:400, Millipore), mouse IgG1 cFos (1:500; Abcam). Secondary antibodies corresponded to goat or donkey anti-chicken, mouse IgG2a, IgG2b, IgG1, rabbit, rat coupled to Alexa Fluor 488, 594, 647 or 405 from Invitrogen (1:500 to 1:1000).

**Culture and tissue imaging**

*Videomicroscopy*

For axonal transport live imaging, cells were grown on 35 mm glass-bottom dishes (81158; Ibidi, BioValley), transfected as described above and imaged at DIV16-17. The culture medium was replaced by a culture medium without phenol red prior to imaging. During live imaging, dishes were placed in a temperature-controlled imaging chamber stabilized at 37°C under 5% CO2. Single channel live imaging was performed on an Axio oberver7 inverted Zeiss microscope using a 63× PlanApo oil objective (numerical aperture = 1.4) and captured using a Hamamatsu Orca-fusion 2 cameras through a mCherry filter set. We used a mercury lamp (Osram HBO 100 W/2). Images were taken distally to the axon initial segment (AIS) for 50s with an exposure time of 150 to 160 ms per image Zen blue 2.6 software (Zeiss).

*Confocal microscopy*

Confocal microscopy was performed using an upright FV-1200 Confocal Microscope and an inverted SP8 Leica confocal microscope, with 40x or 63x oil immersion objectives (1.30 and 1.40 numerical aperture respectively), controlled by Metamorph (FV-1200) or LasX (SP8, Leica, v 3.5.6) softwares. To test the effect of neuronal activity on node-like cluster formation for each acquisition, 387.69 μm x 387.69 μm of 2048x2048 pixels image stacks, with a z-step of 0.35 μm were acquired with the 40x objective using 405, 488, 565 and 647 laser lines. For imaging of the organotypic culture slices, fields of view with Purkinje cells expressing whether mCherry (for chemogenetic experiment) or YFP (for optogenetics experiment) were chosen. A stack sufficient to follow their axons was taken. When imaging to quantify the density of node-like clusters a minimal field of view of 123.14 μm x 123.14 μm of 1024x1024 pixels image stack, including at least 10 Z-series with a z-step of 0.30 μm was acquired with a 63x objective.

In order to quantifiy the density of node-like clusters in mouse spinal cord, we imaged fields in the center of the LPC lesion (5 images per animal). Each acquisition corresponds to a 160.61 μm x 160.61 μm image stack (1024x1024 pixels), including at least 20 images per stack, with a z-step of 0.30 μm. All the images were acquired with an inverted SP8 Leica microscope with a 405, 488, 565 and 647 laser line and a 63x oil immersion objective with a numerical aperture of 1.40, controlled by LasX software (SP8, Leica, v 3.5.6).

All the images generated were blinded before analysis, using a macro developed in the team.

*Myelination index quantification in cultured cerebellar slices*

To evaluate the impact of neuronal activity modulation on myelination, cultured cerebellar slices transduced with AAV8-hSyn-hM3D(Gq)-mCherry were treated for 6 hours with CNO (or DMSO for control condition) at 3 DIV and fixed the following day or L7-Chr2-YFP slices were stimulated with 470nm blue light for 6 hours (versus no stimulation for the control condition) at DIV3 and fixed the following day (17 hours after the end of the stimulation). Stainings were performed to visualize mCherry or YFP expression, as well as Purkinje cells (Calbindin) and myelin (PLP). Five high-resolution images were analyzed per condition per animal (1900 × 1900 pixels, ~550 × 550 μm). The myelination index was computed semi-automatically using a custom ImageJ script. A region of interest focusing on Purkinje cell defasciculated axons (ie excluding soma and white matter tracks) was manually delineated. Binary masks were generated for the total axonal area (Calbindin^+^ pixels) and for the myelinated axonal area (PLP^+^ pixels overlapping with Calbindin^+^ pixels). The myelination index was defined as the ratio of the myelinated axonal area to the total axonal area. The mean index per animal was obtained by averaging the results of the five images.
